# The Current and Retrospective Cognitive Reserve (2CR) survey and its relationship with cognitive and mood measures

**DOI:** 10.1007/s10433-023-00766-x

**Published:** 2023-06-14

**Authors:** Erika Borella, Paolo Ghisletta, Elena Carbone, Stephen Aichele

**Affiliations:** 1 Department of General Psychology, University of Padova, Padova, Italy; 2 Faculty of Psychology and Educational Sciences, University of Geneva, Geneva, Switzerland; 3 UniDistance Suisse, Sierre, Switzerland; 4 Swiss National Centre of Competence in Research LIVES, University of Geneva, Geneva, Switzerland; 5 Department of Human Development and Family Studies, Colorado State University, Fort Collins, CO USA; 6 Department of Epidemiology, Colorado School of Public Health, Fort Collins, CO USA

**Keywords:** Cognitive reserve, Cognitive functioning, Working memory, Depression, Factor analysis

## Abstract

Cognitive Reserve (CR) is often assessed with surveys spanning demographic, lifestyle, and socio-behavioral variables. The role of both past and current life experiences on CR has, however, rarely been examined. We developed the Current and Retrospective Cognitive Reserve (2CR) survey to assess classical CR proxies (socio-economic status, engagement in leisure and social activities) and other dimensions of potential importance (family engagement, religious/spiritual activity) both currently (CRc; in later adulthood) and retrospectively (CRr; as recalled from younger adulthood). We administered the 2CR, measures of general cognitive functioning, working memory (WM), crystallized—vocabulary— and fluid—reasoning—intelligence, and depressive symptoms (DS) to 235 community-dwelling Italian adults (ages 55–90 years). We used exploratory and confirmatory factor analyses to examine the 2CR latent structure, and we estimated correlations of its dimensions with cognitive abilities and DS. Analyses confirmed a three-level factor structure with two global CR factors (CRc and CRr) at the top level, dimensional CR factors (socio-economic status, family engagement, leisure activity, social engagement, and religious/spiritual activity) at mid-level and observed items at the lowest level. Item-factor representations partially differed across CRc and CRr. Both CRc and CRr were positively correlated with measures of intelligence, WM and DS, but associations of measures of intelligence were stronger for CRr, whereas associations of WM and DS were slightly stronger for CRc. The 2CR can be considered a reliable survey for assessing CR proxies within a multidimensional, “life stage-dependent” framework insofar as CRc are CRr closely related but also differently associated with intelligence, WM, and DS.

**Supplementary Information:**

The online version contains supplementary material available at 10.1007/s10433-023-00766-x.

## Introduction

Different compensating factors can counteract/delay cognitive losses linked to the normal aging process and/or to neurodegenerative disorders. One such factor is reserve capacity, i.e., the ability to preserve functionally appropriate behaviors despite the presence of age- and/or pathology-related changes in neurocognitive status. The Cognitive Reserve (CR) model conceives CR as a dynamic and active process, whereby the differential recruitment of cognitive strategies/neural networks underlying task performance allows individuals to cope better with brain damage (Stern 2009). The operationalization of CR is, however, complex, since it cannot be measured directly (Cosentino and Stern 2013). Therefore, CR is usually assessed using proxies based on indicators of lifestyle behaviors and activities that, if “adopted”, serve to protect or augment it.

Commonly used CR proxies include education level, occupational attainment, and engagement in intellectually stimulating leisure activities (Opdebeeck et al. 2016; Stern 2009; 2019). These proxies are thought to reflect a cognitively “enriched” environment, which confer resilience to the neuropathology detrimental effects on functional behaviors and clinical outcomes (Reuter-Lorenz and Park 2014). There is, for example, substantial evidence to show that higher education, along with higher occupational status and engagement in leisure and mental activities, lowers the risk for developing dementia (Opdebeeck et al. 2015; Valenzuela and Sachdev 2006) and can counteract age-related worsening in cognitive abilities and everyday functioning (Ihle et al. 2019).

These CR proxies are frequently examined in isolation and most often limited to education. However, a single indicator is unlikely to capture the full CR dimensionality (Cosentino and Stern 2013). Additionally, assessment with a single indicator does not allow for the statistical differentiation of reliable vs. unreliable sources of variance. Therefore, multi-item surveys thought to reflect the different dimensions of CR have been developed and are increasingly used. These typically include various lifestyle and socio-behavioral items, which are aggregated to provide a global CR score and/or scores for more specific CR dimensions (e.g., leisure engagement encompassing time spent performing intellectually, socially, and/or physically stimulating leisure activities/hobbies; Nucci et al. 2012). However, these inventories do not account for the likelihood that such CR proxies vary across different life course periods (Stern et al. 2019).

To better capture the dynamic nature of CR, it is imperative to assess both overall and domain-specific activities and experiences as they manifest in later life (i.e., as currently assessed in older adults) and also as they have been accumulated over the lifespan (i.e., as retrospectively assessed). To our knowledge, of the extant multidimensional CR surveys, only three assessed CR proxies at different life stages: the Cognitive Reserve Scale [CRS] by Leon et al. (2014) and the Lifetime of Experiences Questionnaire [LEQ] by Valenzuela and Sachdev (2007)—or at specific ages: the Lifetime Cognitive Activity Scale [LCAS] by Wilson et al. (2013).

Notably, the LCAS and CRS do not solicit the classical (and arguably fundamental) CR proxies of education level and occupational status. Moreover, despite a range of intellectually, socially, and physically stimulating activities reflected across these surveys, none include measures of family/relational support or engagement in spiritual/religious activities. Communication and contact with family members and close friends have been shown to contribute both to emotional wellbeing and cognitive functioning in older adults (Ihle et al. 2021; Kelly et al. 2017), and thus to successful/healthy aging (Rowe and Kahn 1998). Moreover, the physical and psychological health of a person’s marital/domestic partner are known to impact both emotional and cognitive outcomes in adulthood (Xu et al. 2016). Spiritual/religious engagement often spans both personal and social dimensions and has been suggested to influence CR as a strengthening factor within the successful aging framework (Hosseini et al. 2019).

Finally, these inventories usually provide a single global score for CR. To our knowledge, no CR survey has thoroughly examined the latent representation/structure of specific CR dimensions within and across current vs. retrospective life stages. By extension, dimensional CR factors assessed at different life stages have not been validated against objective measures of cognitive performance (e.g., working memory, fluid abilities) or closely related affective measures (e.g., depressive symptoms) known to be sensitive to age- and pathology-related declines. This matters because current vs. retrospective CR, and specific dimensions thereof, may be differentially related to such outcomes (Ihle et al. 2021; Rosen et al. 2002).

Here, we present and evaluate a new CR survey, the Current and Retrospective Cognitive Reserve (2CR), aimed at capturing CR as a multidimensional and non-static construct by comprehensively assessing classical and novel CR proxies with respect both to participants’ current status at the time of assessment (CRc) and retrospectively (i.e., as recalled to have occurred in younger adulthood; CRr). The 2CR comprises groups of items related to classical socio-demographics (e.g., educational and occupational attainment, financial status). It also assesses the frequency of engagement in a varied typology of stimulating activities, here organized into leisure activity (encompassing recreational exercise, creative expression, and intellectual stimulation) and social engagement (e.g., volunteering, club membership, public events). Moreover, it is the first to include items related to family engagement (including partnership quality) and spiritual/religious engagement.

We examined CR structure as captured by the survey using confirmatory factor models, testing a three-level latent representation of CR (which was informed by results from a prior exploratory pilot study; see Additional file 1). Specifically, we modeled two global current and retrospective CR factors (CRc and CRr) at the top level, with CR dimensional factors (i.e., socio-economic status, leisure activity, social engagement, family engagement and spiritual/religious engagement) at the intermediate level, and finally with items/parcels at the observed/base level.

We further estimated associations of the global and dimensional CR factors with objective cognitive measures, i.e., general cognitive status, vocabulary and reasoning—for crystallized and fluid intelligence, respectively—and a measure of working memory previously shown to be sensitive to normative aging (Borella et al. 2008). Furthermore, since cognitive impairment and depression are often comorbid in later life (e.g., da Costa Lane Valiengo et al. 2016), we also examined here, for the first time, associations of currently and retrospectively assessed CR factors (global and dimensional) with depressive symptoms (DS).

In general, we expected that the structural factor analyses would support our three-level model, with reliable loadings of survey items onto domain-specific CR factors, and strong domain-specific CR factor loadings onto global CR factors. Moreover, we anticipated moderately positive correlations between current and retrospective factors. Note that results from our pilot study indicated partially differential item representation for the CRc and CRr dimensional factor. This was expected given that goals and resources change across the adult lifespan (Ebner et al. 2006) and is reflect in the factor model underlying the currently tested version of the 2CR.

We further expected modest but significant positive correlations between the CR factors and objective cognitive measures, in line with previous evidence (Opdebeeck et al. 2016) and indicative of broad-domain convergent validity. Because depression impacts mental health outcomes (da Costa Lane Valiengo et al. 2016) and might result in an “impoverished environment” in terms of mental and social stimulation, and given evidence that interactions with loved ones and participation in leisure, social, and religious activities may lower depression risk (Dezutter et al. 2006; Handing et al. 2022), we expected significant negative correlations between the CR factors and DS.

## Methods

### Participants

The sample consisted of 235 individuals over 55 years of age (see Table 1). They were all volunteers, community-dwellers of various Italian cities. None of the participants had a history of psychiatric or neurological disorders, or cognitive difficulties [participants’ Mini-Mental State Examination (Folstein et al. 1975) scores ≥ 27]. None met the criteria for clinical depression [participants’ Geriatric Depression Scale (Yesavage et al. 1983) scores ≤ 5]. The study was approved by the local ethical committee for psychological research.

**Table 1:** Characteristics of the sample

Characteristic	Summary statistic
Total participants	*N* = 235
Women	*n* = 128 (54.5%)
Age in years	*M* = 68.3, *SD* = 8.8, range = 55.0–90.0
Years of education	*M* = 10.2, *SD* = 4.1, range = 4.0–20.0
Partnered (= yes)	*n* = 199 (84.7%)
Children	*M* = 1.8, *SD* = 1.0, range = 0–5
*Occupational category*	
Manual, unqualified	*n* = 47
Manual, qualified	*n* = 78
Non-manual, qualified	*n* = 65
Professional, degreed	*n* = 26
Director or manager	*n* = 19
*Cognition and depression measures*	
MMSE adjusted for age, education (general cognitive functioning)	*M* = 28.4, *SD* = 1.2, NA = 20
WAIS Vocabulary (crystallized intelligence)	*M* = 41.6, *SD* = 10.9, NA = 0
Raven’s total correct (fluid intelligence)	*M* = 31.4, *SD* = 10.0, NA = 1
LST - words recalled (working memory)	*M* = 11.1, *SD* = 3.3, NA = 11
GDS (depressive symptoms)	*M* = 2.0, *SD* = 1.6, NA = 0

MMSE = Mini-Mental State Examination. WAIS = Wechsler Adult Intelligence Scale. LST = Listening Span Task. GDS = Yesavage Geriatric Depression Scale. NA = number of missing observations. There were no missing data for sociodemographic variables

### Materials

#### Current and Retrospective Cognitive Reserve survey (2CR)

The first version of the 2CR included items of key importance from existing CR questionnaires and from an extensive review of literature on healthy aging and cognition. This initial survey (2CR pilot) was administered to a sample of 342 community-dwelling older adults. These pilot data were then factor analyzed and cross-validated against measures of cognitive performance and health status (see Additional file 1). Guided by these—pilot—study results, we developed the current version of the 2CR (Additional file 2), which we administered to a new, independent sample of older participants (described above).

In addition to basic socio-demographic variables (age, sex), the survey comprises items spanning five dimensions of experience: socio-economic status, leisure activity, social engagement, spiritual/religious practice, and family engagement. Except for family engagement, these dimensions were assessed with respect both to current status (i.e., late adulthood/older adulthood) and retrospective status (youth or younger adulthood). We developed the 2CR to be flexible with respect to the age range implied by “retrospective,” i.e., to support different research goals with respect to developmental comparison. For the current study, retrospective referred to younger adulthood, i.e., ages 20–35/40 years^1^. As noted, family engagement was assessed only with respect to current status. This was because associated variables (partnership quality items, e.g., cognitive and emotional status of one’s spouse) would not be applicable if “retrospective” were operationalized as referring to late adolescence.

All response-level items were scaled 0–4, except for educational level, which was scaled from 1 to 7 to cover all of the major educational attainment levels provided by the Italian education and training system (higher scores = higher level of education completed). For the items assessing the engagement in leisure, social, and spiritual/religious activities, participants were asked to rate their frequency of engagement with each of the activities choosing the following options: never, seldom (yearly), sometimes (monthly), often (weekly), always (daily).

**Fig. 1:**
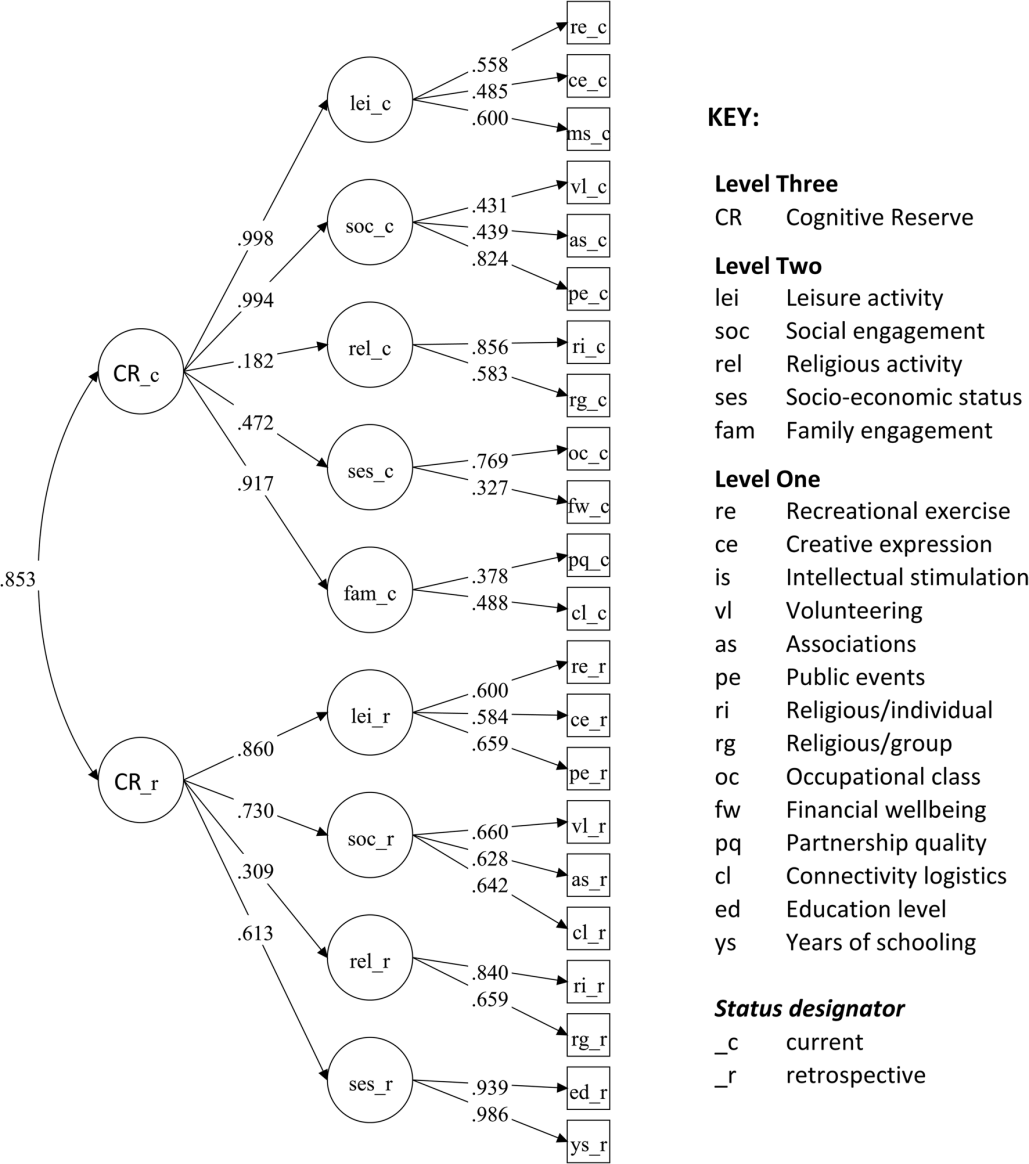
Diagram of the three-level latent structure of the final 2CR survey. *Note*. Standardized factor loadings estimates (shown on directional pathways) were obtained from a three-level factor model, with global CRc and CRr factors at the top level, dimensional CR factors at mid-level, and observed items at the lowest level. Most level one (observed response) items were calculated as composite scores, averaging across response items as follows: recreational exercise (swimming, gym, dance); creative expression (writing, painting, playing music); intellectual stimulation (reading, puzzles, chess); volunteering (at hospitals, schools, charitable organizations); associations (clubs, political groups, professional groups); public events (theatre/concerts, museums, conferences); religious/spirituality-individual (prayer, meditation); religious/spirituality-group (participation in religious rites/ceremonies, church community events); partnership quality (partner’s accessibility, health, mood, mental health); connectivity logistics (driving and computer use related to family/social purposes). Unique item variances and covariances are not shown to allow for a clearer visual presentation of associations between items/factors. Global CRc and CRr factors were correlated at *r* = 0.853, *p* < 0.001

The structure of the 2CR corresponds to a three-level factor model, with general current and retrospective CR (CRc and CRr) at the top level, i.e., at level-3, the above-described CR dimensions as latent constructs at level-2, and observed items at level-1. All level-1 items except for indicators of socio-economic status were obtained as composite scores (parceled as means for this analysis) of sets of two or more items (see Fig. 1 for a diagram of the full model, with composite items further described in the corresponding figure caption and in the scoring sheet for the 2CR in Appendix A).

#### Listening Span Test (LST; Borella et al. 2008)

It consists of sets containing increasing numbers (from 2 to 6) of short sentences. Participants were instructed to listen to each sentence, judge whether it was true or false, and retain its last word. At the end of each set, participants were asked to recall the last word of each sentence. The total number of correctly recalled words was used as a measure of WM performance (maximum score = 20).

#### Wechsler adult intelligence scale revised—vocabulary subtest (Weschler 1981)

A list of 35 words was presented, and participants were asked to provide either their meaning or a synonym. Answers to the 35 items were scored according to the manual. The dependent variable was the sum score for all items (maximum score = 70).

#### Raven’s progressive matrices (Raven’s; Raven et al. 1977)

Participants were presented with 60 matrices. The matrices were similar to a puzzle with a piece missing from the bottom right corner. The participants had to choose which of the 6 pieces presented best completed the figure and to complete the test within a 20-min time constraint. The total number of correct solutions was used as a measure of fluid intelligence.

### Procedure

All participants completed one in-person individual session with an experimenter, lasting about 90 min (with a 5–10-min mid-session break). After obtaining participants’ consent, the order of the tasks/questionnaire was: the MMSE, the 2CR, the Verbal intelligence, the Raven’s, the LST, and the GDS.

### Analyses

We conducted confirmatory factor analyses of the survey data using Mplus statistical software (Muthén and Muthén 2017) with full information maximum likelihood (FIML) estimation and treating response items as continuous variables. We fit three models, beginning with the “full” three-level model (Fig. 1). This model included observed response items at level-1 (a mix of individual item scores and composite/item parcel scores). The level-1 items in turn loaded onto level-2 “dimensional” CR factors, which included five current domains (leisure activity, social engagement, religious activity, socio-economic status, and family engagement) and four retrospective domains (again, family engagement was not assessed retrospectively). Finally, the dimensional level-2 factors loaded onto global CRc and CRr factors at level-3.

Note that, according to the factor-item structure that emerged from the pilot study, relations between some observed activities and dimensional CR factors differed across retrospective vs. current life periods; e.g., connectivity logistics loaded onto social engagement retrospectively, whereas connectivity logistics loaded onto family engagement currently (see Fig. 1).

As for socio-economic status, most people finish formal schooling in adolescence or early adulthood, and education contributes strongly to subsequent occupational experiences (Wilson et al. 2013). Occupational attainment is indeed highly correlated with educational achievement and it -in and of itself- represents a form of lifelong education (an individual's occupation may provide opportunities for cognitive stimulation and new learning) (Scarmeas and Stern 2004). Therefore, we used education to represent retrospective SES, while occupational attainment, along with financial wellbeing, to represent current SES. With respect to retrospective SES, we included both educational level (highest level of education achieved) and years of formal schooling as indicators. As a construct, education level is prone to variation across different types of high schools and/or universities (which may also differ in quality) within and across different countries, potentially influencing scoring equivalence across contexts. Years of schooling better expresses variation in the number of years taken to obtain a given degree, thereby better reflecting differences in normative vs. delayed/accelerated learning. Though related, these two indexes therefore represent two different dimensions of educational attainment, and considering both would allow one to better capture—quantitative and qualitative—sources of educational benefits as a proxy of CR (see Lawrence et al. 2016; McDowell et al. 2007).

In a second model, we only included a single global CR general factor at level three for comparison purposes (i.e., to see if the dimensional factors would collapse onto a single, overarching CR dimension). Finally, we estimated associations between the global and dimensional CR factors and measures of cognitive performance and depressive symptoms. Note that unique variances for level-1 items that were identical save for the distinction of current vs. retrospective were allowed to correlate to account for shared method variance.

## Results

Model fit statistics for the confirmatory factor analyses are provided in Table 2.

**Table 2:** 2CR structural equation model fit statistics

Model	Parameters	χ^2^ (*df*)	CFI	RMSEA	[95%CI]	SRMR	AIC
1. Three-level (CRc, CRr)	88	355 (187)	0.913	0.062	[0.052, 0.072]	0.067	12,606
2. Three-level (CRc + r)^†^	87	369 (188)	0.906	0.064	[0.054, 0.074]	0.068	12,619
3. Two-level (dimensional factors only)	110	312 (165)	0.924	0.062	[0.051, 0.072]	0.061	12,607

CFI = comparative fit index. RMSEA = root mean square error of approximation. SRMR = standardized root mean square residual. AIC = Akaike Information Criterion

^†^Non-positive definite residual covariance matrix (theta)

Overall fit was acceptable across all models based on established cutoff criteria (Hu and Bentler 1999). However, the residual covariance matrix (theta) was non-positive definite for the three-level model with a single global CR factor. A formal test of change in model fit across the three-level models (Δχ^2^ = 14, *df* = 1, p < 0.001) further confirmed selection of the model with two global CR factors (CRc, CRr) over the model with a single global CR factor. Standardized item/factor loadings for the three-level CRc and CRr model are shown in Fig. 1 (additional parameter estimates are provided in Supplemental Materials 2). Standardized loadings (λ) were all strong (≥ 0.400) with a few exceptions. For level-1 items, current financial wellbeing (reverse-coded financial hardship) and family engagement had moderate loadings (λ = 0.327 and λ = 0.378, respectively) onto the corresponding level-2 items. For level-2 items, current and retrospective religious activity loaded weakly/moderately onto the corresponding global CR factors (λ = 0.182 and λ = 0.309, respectively). Global current and retrospective cognitive reserve factors were strongly positively correlated (*r* = 0.853).

### Convergent and discriminant validity

Table 3 shows the correlations of the 2CR global and dimensional factors with variables of interest. With respect to the demographic measures, chronological age was weakly negatively correlated with current leisure activity and social engagement and was weakly negatively correlated with retrospective leisure activity, SES (education), and global CR. Chronological age was moderately negatively associated with current family engagement. Men had weakly/moderately lower religious activity, currently and retrospectively, than women. Men had weakly/moderately higher current SES (occupation) and family engagement, and weakly/moderately higher retrospective social engagement and CR, than women.

**Table 3:** Correlations of 2CR factors with cognitive performance and depressive symptoms

Covariates	Dimensional CR factors									Global CR factors	
	LEI_c	SOC_c	REL_c	SES_c	FAM_c	LEI_r	SOC_r	REL_r	SES_r	CR_c	CR_r
Age	− 0.164	− 0.250	*0.141*	− 0*.003*	− 0.390	− 0.190	− 0*.145*	*0.058*	− 0.312	− 0*.222*	− 0.307
Male^†^	− 0*.057*	*0.005*	− 0.323	0.336	0.316	*0.196*	0.220	− 0.307	*0.144*	*0.067*	*0.218*
MMSE (general cognitive functioning)	− 0*.099*	− 0*.025*	− 0.264	− 0*.011*	*0.196*	− 0*.111*	*0.093*	− *0.030*	*0.073*	− *0.024*	*0.029*
WAIS Vocabulary (crystallized intelligence)	0.376	0.468	*0.054*	0.622	0.221	0.391	0.262	*0.078*	0.600	0.481	0.562
Raven’s (fluid intelligence)	0.367	0.370	− 0.182	0.353	0.376	0.389	0.222	− 0*.105*	0.513	0.409	0.498
LST (working memory)	0.369	0.458	*0.015*	0.530	0.362	0.350	*0.136*	− *0.050*	0.452	0.481	0.435
GDS (depressive symptoms)	− 0.278	− 0.272	− 0*.104*	− 0.316	− 0.595	− 0.219	− 0*.139*	−0 *.121*	− 0.135	− 0.369	− 0.232

Factor abbreviations are defined in Table 1. Factors’ suffix “_c” refers to current, and suffix “_r” refers to retrospective. Correlations between covariates and dimensional CR factors were estimated in a first-order factor model (i.e., without global CR factors). MMSE = Mini-Mental State Examination, adjusted for age and education level. WAIS vocabulary subtest. Raven’s = Raven’s progressive matrices, total correct. LST = listening span task (working memory), words correctly recalled. GDS = Yesavage Geriatric Depression Scale. Non-significant (*p* > .05) correlations are shown *italicized*. The listed covariates were added to models 1 and 3 (Table 2), respectively. This allowed us to calculate correlations of these measures with the global CR factors (model 1) and with the dimensional CR factors (model 3)

^†^Point-biserial correlations

### Associations with cognitive performance measures

Global CRc and CRr factors were not significantly related to MMSE, but both showed moderately strong positive associations with vocabulary (*r* = 0.481 and *r* = 0.562, respectively), reasoning ( *r* = 0.409 and *r* = 0.498), and WM (*r* = 0.481 and *r* = 0.435).

Of the dimensional CR factors, current and retrospective leisure activity, current social engagement, and current and retrospective SES were moderately positively associated with vocabulary (*r* = 0.376–0.622), reasoning (*r* = 0.353–0.513), and WM (*r* = 0.350–0.530). Current family engagement was weakly positively associated with vocabulary (*r* = 0.221) and moderately positively associated with reasoning (*r* = 0.376) and WM (*r* = 0.362). Current religious activity was weakly negatively associated with MMSE scores (*r* = -0.264) and with reasoning (*r* = -0.182). Retrospective religious activity was not significantly associated with any of the cognitive performance measures.

### Associations with depressive symptoms

Of the global CR factors, CRc was moderately negatively associated with depressive symptoms (*r* = − 0.369), and CRr was weakly negatively associated with DS (*r* = − 0.232). Of the dimensional CR factors, current family engagement was most strongly negatively associated with DS (*r* = − 0.595). Current and retrospective leisure activity, current social engagement, and current and retrospective SES were weakly negatively correlated with GDS (*r* = − 0.135–0.316).

## Discussion

We developed the 2CR survey to characterize the dynamic nature of (classical and new) CR proxies in two main life stage periods, i.e., currently (as typically assessed) and retrospectively. We evaluated its factor structure, and results supported a three-level representation of CR, with distinct, global current and retrospective CR factors (CRc and CRr) at level-three, dimensional CR factors at level-two, and with observed items and composite scores at level-one. This depth of representation is, to our knowledge, novel among CR surveys considering different life stages. Global and dimensional CR factors were further examined in relation to objective measures of cognitive ability and, for the first time, depressive symptoms.

### Survey structure and item-factor representation

#### Associations between global (level three) and domain-specific (level two) CR factors

With respect to the 2CR latent structure (Fig. 1), except for family engagement (which, as noted in the Methods, we did not assess retrospectively), the global/domain-specific CR structural sub-models were identical for current and retrospective variables. Loading strengths of the domain-specific CR factors onto the global CR factors were highly consistent, with leisure activity and social engagement most strongly representative of each global factor. Along with socio-economic status, leisure and social domains are not only related to successful/active aging (Rowe and Khan 1998), but they are also well-established socio-behavioral CR proxies (Opdebeeck et al. 2016; Stern et al. 2019), so their salient loadings are reassuring. In contrast, religious/spiritual activity was only weakly associated with both global CR factors, an unexpected result. It may be that religious/spiritual activity is more closely linked to other psychological aspects (e.g., fear of dying; Fortner et al. 2000) than to cognitive protective factors generally. Family engagement also loaded strongly onto CRc, a result consistent with recent evidence on the importance of family (and especially partnership quality) for individual psychological well-being and cognitive functioning in later adulthood (Handing et al. 2022; Kelly et al. 2017).

#### Associations between domain-specific CR factors and observed variables

In contrast to the higher-order (global/domain-specific) CR factor structure, associations between the domain-specific CR factors (level two) and observed/composite items (level one) were mostly asymmetrical across current vs. retrospective variables. As explained in the Methods, asymmetries related to SES and family engagement were inherent to the nature of the domains themselves (e.g., occupational class and partnership/marital status are not relevant when assessing retrospective status as pertaining to late adolescence). Such item-factor structural asymmetries were determined from the pilot study factor analyses (Supplemental Materials 1). For example, connectivity logistics (driving, telecommunications usage) were more closely related to family engagement in the current period (older age), but to general social engagement retrospectively. Similarly, participation in public events was related to social engagement in later adulthood, but to leisure activities in earlier adulthood. These qualitative asymmetries were thus effectively “baked into” the survey in its present form and as administered to the current sample of participants. The results (model fits, factor loadings) from the structural factor analyses applied to these data largely support this structure.

### Associations with cognitive performance

Differential associations were observed between the CR factors (global and dimensional) and cognitive performance measures. Both CRc and CRr were significantly positively correlated with vocabulary and reasoning (measures of crystallized and fluid intelligence), consistent with prior evidence (Opdebeeck et al. 2016). However, associations with these measures were more pronounced with respect to CRr. Both CRc and CRr were similarly both positively associated with WM, but in this case, the association was slightly stronger for CRc. Differences in intelligence, and corresponding differences in educational attainment and behavior-related risk factors for cognitive decline, manifest at an early age and carry forward across the lifespan (Lövdén et al. 2020). In contrast, WM deficits, on average, become increasingly evident in later adulthood, at which time they may begin to interfere with and account for self-care behaviors and social support structures (Borella et al. 2008, 2017). Such developmental patterns are consistent with the observed differential associations of CRc and CRr with objective measures of intelligence and memory.

Neither CRc nor CRr correlated with general cognitive status (MMSE). Basic assessments of cognitive status (e.g., awareness of the day and date) are commonly used to evaluate neurological functioning during clinical intake, but these comparatively coarse measures may lack sensitivity to CR dimensions that track across a more varied range of individual ability (Arcara et al. 2017). Associations between dimensional CR factors and objective cognitive performance were most pronounced with respect to SES (current and retrospective). Especially salient relations were observed between SES (current and retrospective) and crystallized intelligence, confirming that an “enriched” learning environment, in terms of educational and occupational stimuli and opportunities, may promote/sustain crystallized intelligence (Cheng and Furnham 2019).

There were also differentiated relations between leisure activity (current and retrospective), social engagement (current), and family engagement (current) dimensional CR factors and the objective cognitive measures. Notably, whereas leisure activity, social engagement (current), and SES CR factors were most strongly linked to crystallized intelligence (which remains relatively stable across the adult lifespan), family engagement was more strongly linked with reasoning and WM. This result is consistent with studies showing WM and fluid abilities as playing important roles for engaging in social activities and close interpersonal relations (Kelly et al. 2017). Reciprocally, there is also evidence linking better socio-relational functioning (as a precursor) to reduced age-related loss in fluid abilities (as outcomes), with cognitive reserve playing a mediating role (Ihle et al. 2021).

Current religious activity was negatively (albeit weakly) associated with MMSE scores and with reasoning, whereas retrospective religious activity was not significantly associated with any of the cognitive performance measures. Although this result was not anticipated, prior studies have similarly shown that religiosity is negatively correlated with fluid reasoning abilities (Daws and Hampshire 2017). This result may reflect how such religious/spiritual activities were assessed on the 2CR, and it may also reflect the specific population from whom the current data were obtained. These are certainly considerations for evaluating such associations in different cultural contexts and age groups in future studies using the 2CR, for which religious/spiritual practice may have a protective effect. Indeed, religious/spiritual practice may differentially manifest as a function of age across cultural settings, e.g., because some cultures may afford more opportunities for (or place stronger expectations on) older adults to be religiously observant.

### Associations with depressive symptoms

As expected, moderately strong negative correlations were observed between DS and global CRc, family engagement, and current SES. DS were also weakly negatively associated with leisure activity (current and retrospective), social engagement (current), SES (retrospective), and CRr. The result that family engagement was most strongly associated with reduced DS is consistent with recent studies showing that social isolation, particularly related to increased physical distance from loved ones (consistent with our indicators for family engagement) is a potent risk factor for depression in older adults (Handing et al. 2022). Increased social selectivity (smaller social networks, more narrowly prescribed social activities) during adulthood may serve to ensure more positive social exchanges (Carstensen 1995; Luong et al. 2011) but may also place increased value on such social support for maintaining emotional wellbeing in later life.

### Limitations

An important limitation of this study related to sample selection is that study participants were on average cognitively well-functioning, which may further explain the lack of association between 2CR and MMSE scores, and without clinically diagnostic DS. We also lacked neuroimaging data, which would have allowed us to further test CR as a moderator of associations between brain status and cognitive performance. These issues should be examined in future studies to show that better CR, as evaluated on the 2CR survey, better predicts overall functioning in participants with clinically meaningful levels of brain injury and/or dementia.

The survey itself also has some potential limitations. For the pilot study, family engagement indicators also included number of family members (a count of spouse/children/grandchildren); however, we omitted this indicator in the current 2CR model (Fig. 1) due to variable/data characteristics.^2^ We did, however, retain the original items (marital status, number of children and grandchildren) on the final survey for use by others should they desire further validation. We also conducted a follow-up sensitivity analysis including this variable (as a continuous outcome), but the model failed to converge.

In assessing communication, we focused on purpose rather than modality. We therefore combined computer, tablet, and phone use in a single item, as they can all be used for the same goal of communicating with others. Generational differences in device usage patterns may to some extent account for the asymmetry in how this communication item loaded onto current vs. retrospective CR factors, but we believe the 2CR model and survey structure does capture important differences of communication purposes (more broadly social for youth/younger adults, more family-focused for older adults, coherent with Carstensen’s 1995, lifespan theory of socioemotional selectivity; English and Carstensen 2014) and have therefore chosen to retain this as a single item.

Retrospective SES was indicated by two items (education level and years of schooling). Due to the very strong correlation (*r* = 0.92) between these items, we subsequently tested two models wherein retrospective SES was operationalized as either of these observed (singular) education variables rather than as a factor loading on both. However, both models failed to converge. In follow-up checks, we identified 27% of participants as displaying a discrepancy between years of schooling and education level, with approximately 55% of those individuals showing deferred attainment (e.g., due to repeating grade levels, dropping out, or simply due to differences across educational systems). The remainder evinced precociousness (early advancement). In the end, we retained SES as a factor indicated by both education variables (a) for enhanced reliability given the latent-variable definition and (b) for potentially improved validity by accounting for the above-noted discrepancies across education measures.

Finally, we included a variety of common daily life leisure activities known to prompt CR, but these lists were necessarily non-exhaustive. For example, with respect to recreational exercise, three items loaded most strongly (going to the gym, dance, swimming/water aerobics). Notably, these activities concurrently promote aerobic and motor-coordination skill development, and they may also promote social interaction. Such multifaceted exercises may be key to supporting cognitive and mental health in later life (Verghese et al. 2003). That said, researchers using this survey in the future may wish to consider other recreational exercise activities (e.g., walking, cycling)—as well as additional items related to creative expression and “intellectual stimulation.” We therefore have included open-ended response options for each of these survey questions.

## Conclusions

Overall, these findings highlight the life stage-dependent nature of CR, which likely shifts both qualitatively and quantitatively as adults adapt and develop in response to changing demands, goals, and priorities across the lifespan (Ebner et al. 2006). Though CRc and CRr were strongly positively correlated, comparison of model fit clearly favored a solution with two separate global CR factors, compared to a single CR factor, and this was further supported by differential associations of CRc vs. CRr with the objective measures of cognitive performance. It follows that it is important to evaluate CR as a multidimensional and dynamic construct with respect both to the individual’s status at the time/life period of assessment and also in relation to earlier life activities, as reflected in the 2CR survey.

An important dimension of CR not included in other currently available CR inventories is family engagement (encompassing partnership quality), which we found to be significantly positively associated with reasoning and WM performance and negatively associated with DS. This is an especially salient outcome given that family engagement (and partnership quality) has rarely been examined as a CR dimension and given that both cognitive impairment and depression have represented primary concerns for mental health within the older adult population (World Health Organization 2017). Physical proximity to loved ones is likely an essential protective factor for mental health and everyday functioning in later adulthood (Carr and Utz 2020; Handing et al. 2022), so it follows that this dimension merits inclusion as a dimension of CR.

The relationship between CR and cognitive processes central in aging (WM, reasoning and crystallized intelligence) were also confirmed, with evidence of differential associations across CRc (memory was comparatively salient) and CRr (intelligence was comparatively salient), consistent with previous findings across the adult lifespan (e.g., ). Further, we showed for the first time that depressive symptoms were significantly negatively associated with dimensional and global CR factors.

In conclusion, this study shows our new 2CR survey to be a psychometrically sound measure of CR, sensitive not only to differentiated life experiences in early vs. later adulthood, but also to their associations with current cognitive and psychological outcomes in adulthood and older age. The 2CR will likely be useful in clinical practice and could easily be extended also as a form of semi-structured interview regarding individuals’ past and current lifestyle habits. The 2CR may further prove useful for applied research to develop strategies/solutions (social policies, assessment/monitoring, training programs) targeting cost-effective lifestyle factors for improved mental health and quality of life in later adulthood.

## Supplementary Information

Below is the link to the electronic supplementary material.

**Additional file 1.** 2CR data.

**Additional file 2.** 2CR survey protocol.

**Additional file 3.** Mplus output.

## Data Availability

The data that support the findings of this study, the analytic methods, and study materials are available from the corresponding authors upon request.

## Footnotes

1 Note that the survey allows for the age range associated with retrospective status to be specified by the experimenter at the time of administration, e.g., contingent on research goals and sample demographics.

2 Counts of family members were highly skewed, requiring statistical treatment as a categorical variable. For the revised survey used here, we sought to treat all response variables as continuous rather than categorical variables (a) to facilitate model specification/convergence and (b) to support FIML estimation, which accommodates missing data.
