# Supplementary material for: The Current and Retrospective Cognitive Reserve (2CR) survey and its relationship with cognitive and mood measures
Source: Eur J Ageing. 2023 Jun 14;20(1):23. doi: 10.1007/s10433-023-00766-x (PMC10267060; doi:10.1007/s10433-023-00766-x)
Supplement: Supplementary file 1 — Additional file 1. 2CR data. [file 10433_2023_766_MOESM1_ESM.docx]

## Supplemental Materials 1

**The Current and Retrospective Cognitive Reserve (2CR) Survey and its relationship with cognitive and mood measures.**

## 1. Preliminary Factor Analyses for the Current and Retrospective Cognitive Reserve (2CR) Survey with Pilot Data

**1.1 Participants and Split-Sample Methodology**

Data for the initial survey came from 342 Italian-born community-dwelling older adults (mean age = 65.5 years, range = 40–91 years; 51.2% women). For the factor analyses, we used a split sample methodology wherein random sampling without replacement was used to assign participants to one of two groups (G1, G2). Sample size was the same for both groups (n = 171), and sample composition was similar: For G1, participants were 52.6% women, mean age = 65.5 years (range = 41–90 years), and for G2, participants were 49.7% women, mean age = 65.4 years (range = 40–87 years). Data from G1 participants were used for both exploratory and confirmatory factor analyses, whereas data from G2 were used only in confirmatory factor analyses (as described below).

**1.2 2CR Survey Items and Data Pre-Processing**

The original 2CR survey was administered in Italian (an outline of items and original response/scaling protocol is provided in English as Supplemental Materials, S1). Items on the survey spanned multiple domains: education, occupation, financial situation, activities and hobbies (non-sports), exercise and recreational sports, social life (participation in clubs, going out for entertainment, volunteering), religious activity (prayer, church groups, etc.), and family situations (children, partnership quality, etc.). The survey also included health and lifestyle related variables (e.g., smoking status, self-rated nutrition), as well as medical conditions (for validation purposes only).

Data were complete except for single (unpartnered, unmarried) persons’ responses on items specific to partnership (e.g., “How do you perceive your partner’s mood?”). Therefore, partnership related items were combined as summary scores related to (a) scope of family (1 point each if partnered, with children, and/or with nephews/nieces) and (b) partnership quality (subjective quality, partner’s engagement, partner’s health level, partner’s mood). Many items’ response levels were structured according to frequency of activity (e.g., daily, several times per week, monthly, etc.). Items included in the factor analyses were all based on ordinal scaling (most with five levels, some with slightly fewer or more levels).

**1.3 Exploratory Factor Analysis**

We used exploratory factor analyses (EFA) to examine the latent dimensionality of items on the 2CR survey in data for G1. Separate EFA were carried out for items pertaining to current status (older adult; 43 items) and retrospective status (younger adult; 30 items), respectively. All items were treated as ordinal (rather than continuous) variables. We first used parallel analysis (Horn, 1965; Velicer & Jackson, 1990) as applied to Spearman rank-order correlations for estimating the number of underlying factors (latent dimensions). We then conducted EFA using Mplus statistical software (Muthén & Muthén, 2017) with robust weighted least squares estimation and Crawford-Ferguson oblique rotation (kappa = 0.3; Browne, 2001).

1.4 Results

Parallel analyses (Figure S2a) showed that up to five factors could be extracted from the current status items and that up to three factors could be extracted from retrospective status items (i.e., as indicated by observed eigenvalues distinctly larger in magnitude than artificially generated eigenvalues). We therefore extracted these numbers of factors, ±1 factor, for each group of items (thus, there were three factor analyses per group of items). Looking at the resulting factor-item representations, we felt that the original numbers of factors (five and three, respectively) were the best compromise with respect to model fit and interpretation. We assessed model adequacy based on the following cutoff values of three absolute fit criteria: root mean square error of approximation (RMSEA) < .10 (Kenny, Kaniskan, & McCoach, 2015), comparative fit index (CFI) ≥ .90 (Bentler, 1990), and standardized root mean square residual (SRMR) < .80 (Hu and Bentler, 1999). Fit statistics for current status items with five factors extracted were: Х^2^(698) = 894; RMSEA = .04 [.03, .05], CFI = .90, and SRMR = .09. Fit statistics for retrospective status items with three factors extracted were: Х ^2^(348) = 453; RMSEA = .04 [.03, .05], CFI = .89, and SRMR = .09. Standardized factor loadings for current status items and for retrospective status items are reported in Tables S2a and S2b, respectively.

Factors for current status items indicated constructs related to leisure activity (Factor 1c): recreational exercise, creative expression, games; social engagement (Factor 2c): volunteering, association memberships, public events; and religious activity (Factor 3c): church events, meditation, choir, etc.; socio-economic status (Factor 4c): work, education, and financial wellbeing; and family engagement (Factor 5c): which in addition to partnership quality and family size included items such as driving and computer use for visitation and communication. Factor representation for retrospective items was similar, albeit with slight differences, and with constructs indicative of retrospective leisure activity (factor 1r): recreational exercise, creative expression, and attendance of public events; retrospective social engagement (factor 2r): volunteering, association membership, driving and computer use presumably for socializing purposes); and retrospective religious activity (factor 3r).


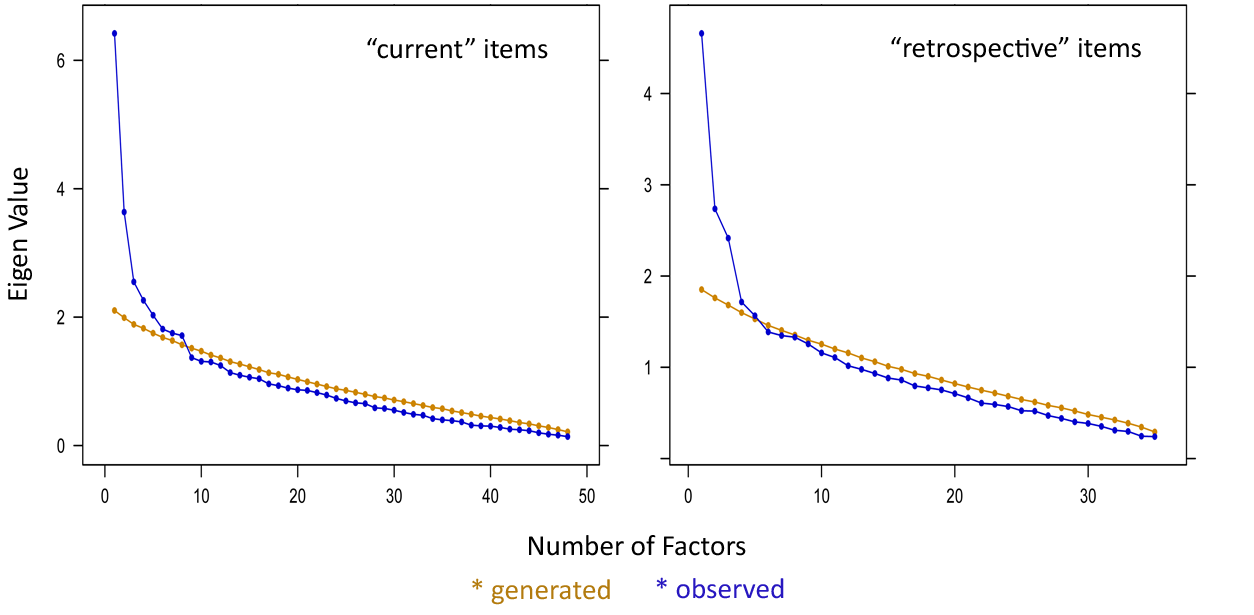


**Figure S1a**. Parallel analysis to decide on the number of factors to extract from observed data corresponding to current and retrospective 2CR survey items. Observed eigenvalues (blue) clearly larger in magnitude than generated eigenvalues (orange) indicate the number of corresponding factors to be extracted from participant data.

## Table S1a. Factor Loadings for Current Status Items on the 2CR Survey

|  | Standardized Factor Loadings | | | | | | | | |
| --- | --- | --- | --- | --- | --- | --- | --- | --- | --- |
| Indicator | Factor 1c | Factor 2c | | Factor 3c | | Factor 4c | | Factor 5c | |
| 01. physical work | -0.23 | 0.18 | | -0.05 | | **0.75** | | 0.03 | |
| 02. occupational class | 0.15 | 0.00 | | 0.14 | | **0.74** | | 0.20 | |
| 03. years education | 0.25 | -0.03 | | -0.01 | | **0.72** | | **0.31** | |
| 04. mental work | -0.22 | 0.07 | | -0.02 | | **0.64** | | 0.03 | |
| 05. financial wellbeing | 0.11 | 0.01 | | 0.01 | | **0.45** | | 0.00 | |
| 06. organized work | 0.05 | -0.02 | | 0.08 | | **0.39** | | 0.23 | |
| 07. creative work | 0.14 | -0.07 | | -0.06 | | **0.36** | | -0.04 | |
| 08. nutrition | 0.09 | -0.15 | | 0.24 | | **0.31** | | -0.28 | |
| 09. playing a musical instrument | **0.59** | -0.09 | | 0.04 | | 0.04 | | 0.20 | |
| 10. painting | **0.58** | **0.42** | | -0.07 | | -0.02 | | -0.05 | |
| 11. reading | **0.52** | 0.01 | | 0.01 | | 0.22 | | 0.15 | |
| 12. writing | **0.52** | 0.00 | | 0.05 | | 0.15 | | 0.09 | |
| 13. swimming | **0.51** | -0.17 | | -0.16 | | -0.03 | | 0.22 | |
| 14. second language | **0.45** | -0.02 | | -0.02 | | 0.27 | | **0.36** | |
| 15. playing chess | **0.45** | -0.27 | | 0.08 | | **0.34** | | 0.10 | |
| 16. crosswords, puzzles | **0.33** | 0.22 | | 0.17 | | 0.15 | | 0.23 | |
| 17. gym | **0.38** | 0.26 | | 0.21 | | -0.12 | | -0.16 | |
| 18. dance | **0.38** | 0.13 | | 0.23 | | -0.19 | | 0.03 | |
| 19. partner quality | -0.23 | -0.21 | | 0.00 | | -0.11 | | **0.78** | |
| 20. driving | 0.05 | 0.09 | | -0.04 | | 0.03 | | **0.74** | |
| 21. computer use | 0.18 | 0.07 | | -0.06 | | 0.24 | | **0.57** | |
| 22. family members | -0.14 | -0.21 | | 0.12 | | -0.12 | | **0.53** | |
| 23. cycling | 0.00 | **0.40** | | -0.15 | | -0.27 | | **0.43** | |
| 24. conferences, public events | 0.03 | **0.78** | | -0.03 | | 0.07 | | 0.09 | |
| 25. exhibition, museums | **0.33** | **0.66** | | 0.16 | | 0.22 | | -0.07 | |
| 26. volunteer for charities | 0.02 | **0.59** | | 0.10 | | 0.08 | | 0.19 | |
| 27. volunteer at hospitals, care facilities | 0.08 | **0.58** | | **0.33** | | -0.05 | | 0.18 | |
| 28. cinema, theatre, concerts | 0.25 | **0.52** | | 0.00 | | 0.09 | | 0.16 | |
| 29. political groups or associations | **-0.40** | **0.52** | | -0.01 | | 0.20 | | **0.31** | |
| 30. senior clubs and associations | **0.40** | **0.47** | | **0.32** | | 0.00 | | -0.05 | |
| 31. walking stairs | 0.09 | **0.43** | | -0.06 | | -0.03 | | 0.06 | |
| 32. walking | 0.11 | **0.35** | | 0.09 | | -0.04 | | -0.24 | |
| 33. religious activity (other) | -0.15 | -0.18 | | **0.85** | | -0.10 | | 0.20 | |
| 34. religious activities (social) | **-0.30** | 0.06 | | **0.77** | | -0.06 | | -0.08 | |
| 35. meditation, chanting, religious singing | -0.03 | -0.18 | | **0.74** | | 0.01 | | -0.28 | |
| 36. religious rites | 0.28 | 0.08 | | **0.63** | | 0.20 | | -0.15 | |
| 37. volunteer at schools, youth facilities | 0.13 | 0.29 | | **0.50** | | 0.12 | | 0.17 | |
| 38. religious reading | 0.24 | -0.11 | | **0.39** | | 0.11 | | 0.01 | |
| 39. professional associations | 0.11 | -0.07 | | 0.10 | | 0.28 | | 0.23 | |
| 40. smoking | -0.16 | -0.03 | | -0.02 | | 0.27 | | -0.25 | |
| 41. subjective health | 0.12 | -0.11 | | -0.07 | | 0.21 | | 0.07 | |
| 42. gardening | 0.15 | 0.08 | | 0.21 | | -0.09 | | -0.09 | |
| 43. playing cards | 0.19 | 0.19 | | 0.24 | | -0.20 | | -0.19 | |
|  | | | | | | | | | |
| ***Interfactor Correlations*** | **Factor 1c** | | **Factor 2c** | | **Factor 3c** | | **Factor 4c** | |  |
| **Factor 2c** | .225 | |  | |  | |  | |  |
| **Factor 3c** | .147 | | .124 | |  | |  | |  |
| **Factor 4c** | .219 | | .078 | | .074 | |  | |  |
| **Factor 5c** | .118 | | .102 | | -.077 | | .214 | |  |

*Note*: Standardized factor loadings with magnitude ≥ .30 are shown in bold.

## Table S1b. Factor Loadings for Retrospective Status Items on the 2CR Survey

|  | Standardized Factor Loadings | | |
| --- | --- | --- | --- |
| Indicator | Factor 1r | Factor 2r | Factor 3r |
| 01. exhibition, museums | **0.79** | 0.14 | 0.14 |
| 02. cinema, theatre, concerts | **0.67** | -0.04 | -0.21 |
| 03. dance | **0.54** | -0.19 | -0.03 |
| 04. gym | **0.54** | 0.05 | 0.07 |
| 05. conferences, public events | **0.53** | 0.14 | 0.08 |
| 06. painting | **0.41** | 0.23 | -0.03 |
| 07. writing | **0.41** | **0.30** | 0.00 |
| 08. playing a musical instrument | **0.40** | **0.33** | -0.07 |
| 09. walking | **0.39** | **-0.36** | 0.29 |
| 10. reading | **0.35** | **0.46** | 0.06 |
| 11. cycling | **0.34** | -0.05 | -0.14 |
| 12. playing chess | **0.31** | **0.47** | 0.01 |
| 13. swimming | **0.30** | 0.21 | -0.13 |
| 14. driving | -0.06 | **0.65** | -0.09 |
| 15. professional associations | 0.04 | **0.55** | -0.03 |
| 16. volunteer at hospitals, care facilities | -0.04 | **0.55** | **0.33** |
| 17. volunteer for charities | -0.06 | **0.54** | 0.25 |
| 18. crosswords, puzzles | 0.15 | **0.44** | 0.11 |
| 19. volunteer at schools, youth facilities | 0.07 | **0.44** | **0.33** |
| 20. computer use | -0.02 | **0.44** | -0.05 |
| 21. political groups or associations | -0.09 | **0.33** | 0.18 |
| 22. gardening | 0.14 | **-0.30** | 0.25 |
| 23. meditation, chanting, religious singing | -0.12 | -0.22 | **0.82** |
| 24. religious activity (other) | -0.14 | 0.11 | **0.70** |
| 25. religious rites | 0.25 | 0.12 | **0.62** |
| 26. senior clubs and associations | 0.25 | 0.25 | **0.59** |
| 27. religious activities (social) | -0.23 | -0.02 | **0.56** |
| 28. religious reading | 0.21 | 0.16 | **0.43** |
| 29. playing cards | 0.13 | -0.03 | **0.32** |
| 30. walking stairs | 0.25 | -0.27 | **0.30** |
|  |  |  |  |
| ***Interfactor Correlations*** | **Factor 1r** | **Factor 2r** |  |
| **Factor 2r** | .222 |  |  |
| **Factor 3r** | .093 | .144 |  |

*Note*: Standardized factor loadings with magnitude ≥ .30 are shown in bold.

**2. Confirmatory Factor Analysis**

Guided by results from the EFA described above, we derived separate structural factor models for items related to current status (five factors) and for items related to retrospective status (three factors). Some of the items for the confirmatory analyses were derived as composite scores (sums) of items on the original questionnaire:

**Composite Items**

1. number of relations (partnered, children, nieces/nephews)
2. partnership quality (subjective quality, partner’s engagement/health/mood)
3. connectivity logistics (driving, computer use)
4. recreational exercise (swimming, gym, dance)
5. creative expression (writing, painting, playing music, gardening)
6. mental stimulation (reading, puzzles, chess, cards)
7. volunteering (hospitals, schools, charities)
8. associations (clubs, political, professional)
9. public events (theatre/concerts, museums, conferences)

Note that two of the items (connectivity logistics, public events) loaded onto different factors in current vs. retrospective models. The factor/item associations can be inferred from Table S2d below.

Under the split-sample methodology, we estimated confirmatory factor models for current and retrospective items within both groups (G1, G2) under configural, strong, and strict factorial invariance (Millsap, 2011; Widaman & Reise, 1997). In total then, we conducted six multiple-group factor analyses (i.e., current status and retrospective status models, each estimated at three levels of measurement invariance, across G1 and G2). Confirmatory factor models were again estimated using Mplus (Muthén & Muthén, 2017) with robust weighted least squares estimation and theta parameterization. The “current status” model included five factors, each represented by three items. The “retrospective status” model included three factors, each represented by three items. When items corresponding to a given factor were all composites, they were treated as continuous variables; otherwise, items were treated as categorical.

2.1 Results

Model fit statistics are provided in Table S2c, and these gave evidence of overall acceptable fit for all models based on established criteria (Bentler, 1990; Hu & Bentler, 1999; Kenny, Kniskan, & McCoach, 2015). Comparatively, results supported strict factorial invariance for models based both on current and retrospective items, meaning that all parameters were equal (invariant) across the two random subsamples (G1, G2). Factor loadings and factor correlations (estimated from the groups’ pooled data) are provided in Table S2d. Standardized factor loadings were all well above .40. The strongest (positive) correlations were observed between leisure activity and social engagement factors. Religious activity was negatively (weakly/moderately) correlated with socio-economic status and family engagement factors. Socio-economic status was positively (moderately/strongly) correlated with leisure activity and social engagement (current and retrospective). Results from a follow-up analysis that included both current and retrospective items showed that corresponding current/retrospective factors were positively (moderately/strongly) correlated.

**Table S1c. Fit Statistics for Multiple-Group Confirmatory Factor Models Under Different Invariance Constraints**

| Factor Model | Invariance | Х^2^ (*df*) | CFI | SRMR | RMSEA [95%CI] |
| --- | --- | --- | --- | --- | --- |
| Current Status Items | configural | 287 (160) | .909 | .076 | .068 [.055, .081] |
|  | strong | 319 (197) | .912 | .079 | .060 [.048, .072] |
|  | strict | 340 (212) | .908 | .082 | .060 [.048, .071] |
| Retrospective Items | configural | 71 (48) | .938 | .053 | .053 [.022, .078] |
|  | strong | 93 (69) | .934 | .059 | .045 [.016, .067] |
|  | strict | 104 (78) | .929 | .064 | .044 [.016, .065] |

*Note*: Strict factorial invariance model was supported for both current and retrospective items.

**Table S1d. 2CR Item-Factor Loadings and Inter-factor Correlations**

|  | Standardized Factor Loadings | | | | |
| --- | --- | --- | --- | --- | --- |
|  | Current Status | |  | Retrospective | |
| FACTOR / indicators | Est. | (SE) |  | Est. | (SE) |
| F1c, F1r: LEISURE ACTIVITY |  |  |  |  |  |
| i1, i1r recreational exercise | .739 | (.042) |  | .637 | (.053) |
| i2, i2r creative expression | .537 | (.050) |  | .569 | (.051) |
| i3 mental stimulation | .612 | (.042) |  |  |  |
| i3r public events |  |  |  | .747 | (.054) |
|  |  |  |  |  |  |
| F2c, F2r: SOCIAL ENGAGEMENT |  |  |  |  |  |
| i4, i4r volunteering | .809 | (.038) |  | .637 | (.053) |
| i5, i5r associations | .558 | (.043) |  | .569 | (.051) |
| i6 public events | .671 | (.043) |  |  |  |
| i6r connectivity logistics |  |  |  | .747 | (.054) |
|  |  |  |  |  |  |
| F3c, F3r: RELIGIOUS ACTIVITY ^†^ |  |  |  |  |  |
| i7, i7r singing/chanting/meditation | .704 | (.061) |  | .714 | (.063) |
| i8, i8r church social events | .765 | (.062) |  | .652 | (.058) |
| i9, i9r other religious activity | .681 | (.060) |  | .686 | (.060) |
|  |  |  |  |  |  |
| F4c: SOCIO-ECONOMIC STATUS ^†^ |  |  |  |  |  |
| i10 occupational class | .910 | (.036) |  |  |  |
| i11 financial wellbeing | .851 | (.035) |  |  |  |
| i12 years of education | .526 | (.057) |  |  |  |
|  |  |  |  |  |  |
| F5c: FAMILY ENGAGEMENT ^†^ |  |  |  |  |  |
| i13 number of family relations | .528 | (.068) |  |  |  |
| i14 partnership quality | .885 | (.090) |  |  |  |
| i15 connectivity logistics | .465 | (.064) |  |  |  |

| ***Inter-factor Correlations*** | **F1c** | **F2c** | **F3c** | **F4c** | **F5c** | **F1r** | **F2r** |
| --- | --- | --- | --- | --- | --- | --- | --- |
| **F2c** | .794 |  |  |  |  |  |  |
| **F3c** | .041 | .085 |  |  |  |  |  |
| **F4c** | .520 | .490 | -.140 |  |  |  |  |
| **F5c** | -.001 | .005 | -.201 | .190 |  |  |  |
| **F1r** | *.789* | *.684* | *-.097* | *.563* | *.250* |  |  |
| **F2r** | *.298* | *.380* | *-.131* | *.706* | *.587* | .452 |  |
| **F3r** | *-.067* | *.004* | *.841* | *-.047* | *-.173* | -.115 | .081 |

*Note*: Factor loadings and factor correlations were estimated independently for models (a) based on current status items and (b) based on retrospective items. An exception to this was for correlations between current and retrospective factors (shown italicized): These were estimated in a model with both current and retrospective factors/items included. The above estimates were obtained from data pooled across the two split-samples (G1, G2) after strict factorial invariance had been established.

^†^ Items for this factor were specified as categorical for estimation purposes.

**3. Follow-up Cross-validation**

Following completion of the 2CR survey, participants also provided information about disease symptoms (current and retrospective; symptoms were reverse coded to indicate health and are listed in Supplement S1, section 9; these were then summed as singular scores), subjective health status, and depressive symptoms as reported on the Geriatric Depression Scale (GDS; Yesavage, Bruik, & Rose, 1983). They also completed several cognitive assessments: the Mini-Mental State Exam (MMSE, Folstein, Folstein, & McHugh, 1975), a measure of general cognitive functioning, the Raven’s Matrices, a measure of abstract reasoning-fluid intelligence (Raven, 1938), and a measure of crystallized intelligence based on vocabulary (Orsini and Pezzuti, 2013, 2015).

Correlations between these measures and the 2CR factors are provided below in Table S2e. SES, Family, Social, and Leisure factors were associated with better cognitive performance and lower depressive symptoms. SES, Leisure, and Social were also associated with better current health. Subjective health was positively associated with SES but other associations were not significant. Religious activity, both retrospectively and currently, was negatively associated with abstract reasoning (Raven’s matrices).

**Table S1e. Correlations of 2CR factors with Other Measures**

| Covariates: | 2CR Factors | | | | | | | |
| --- | --- | --- | --- | --- | --- | --- | --- | --- |
|  | LEISURE | SOCIAL | RELIGION | SES | FAMILY | LEISURE-R | SOCIAL-R | RELIGION-R |
| Age | **0.29** | **0.21** | **0.22** | -0.03 | **-0.47** | **0.13** | **-0.30** | 0.09 |
| Male | -0.02 | 0.04 | -0.09 | 0.06 | **0.36** | 0.11 | **0.36** | **-0.15** |
| MMSE | **0.29** | **0.31** | -0.11 | **0.42** | **0.46** | **0.31** | **0.43** | -0.12 |
| Vocabulary | **0.22** | **0.15** | -0.04 | **0.60** | **0.26** | **0.18** | **0.47** | -0.03 |
| Raven’s | 0.05 | **0.23** | **-0.26** | **0.39** | **0.58** | **0.19** | **0.47** | **-0.15** |
| GDS | **-0.30** | **-0.24** | 0.06 | **-0.19** | **-0.24** | **-0.28** | **-0.31** | 0.06 |
| Health | **0.18** | **0.20** | -0.02 | **0.14** | 0.10 | **0.13** | **0.20** | -0.10 |
| Health-R | 0.10 | 0.01 | -0.01 | -0.01 | -0.02 | -0.05 | -0.07 | -0.03 |
| Subj. Health | 0.01 | 0.01 | -0.09 | **0.15** | 0.08 | 0.05 | 0.07 | -0.12 |

*Note*: “-R” refers to retrospective factors. Significant (*p* < .05) correlations are bolded.

**4. The Final 2CR survey**

In the light of the results from the pilot study, the structure of the 2CR was refined as follow: (a) re-scaled items for increased consistency of response and analytical efficiency, (b) more clearly identified latent dimensions that both did and did not overlap across current and retrospective domains, (c) shortened the survey by removing items that provided little informative value with respect to the identified latent dimensions, and (d) restructured some survey items to include what were previously multiple, conceptually-related items that, individually, offered little advantage for latent construct representation.

These changes resulted in a final 2CR survey (see Appendix A for the protocol and the scoring sheet), with clearer dimensionality, improved scaling, and less redundancy (for improved efficiency of delivery and response.

**References**

Bentler, P. M. (1990). Comparative fit indexes in structural models. *Psychological Bulletin*, *107*, 238–246.

Browne, M. W. (2001). An overview of analytic rotation in exploratory factor analysis. *Multivariate Behavioral Research*, *36*, 111–150.

Folstein, M., Folstein, S., & McHugh, P. (1975). “Mini-Mental State” A practical method for grading the cognitive state of patients for the clinician. *Journal of Psychiatric Research*, *12*, 189–198.

Horn, J. L. (1965). A rationale and test for the number of factors in factor analysis. *Psychometrika*, *30*, 179–185.

Hu, L., & Bentler, P. M. (1999). Cutoff criteria for fit indexes in covariance structure analysis: Conventional criteria versus new alternatives. *Structural Equation Modeling, 6,*1–55.

Kenny, D. A., Kaniskan, B., & McCoach, D. B. (2015). The performance of RMSEA in models with small degrees of freedom. *Sociological Methods & Research*, *44*, 486–507.

Millsap, R. E. (2011). *Statistical approaches to measurement invariance*. Taylor and Francis Group: New York.

Muthén, L. K., & Muthén, B. O. (1998–2017). *Mplus user's guide* (8th ed.). Los Angeles, CA: Muthén & Muthén.

Orsini, A., & Pezzuti, L. (2013). *WAIS-IV. Contributo alla taratura italiana (16–69) [WAISIV, contribution to the Italian standardization, ages 16–69]*. Firenze, Italy: Giunti OS.

Orsini, A., & Pezzuti, L. (2015). *WAIS-IV. Contributo alla taratura italiana (70–90 anni) [WAIS-IV, contribution to the Italian standardization, ages 70–90]*. Firenze, Italy: Giunti OS

Raven, J. C. (1938). *Raven's progressive matrices*. Los Angeles, CA: Western

Velicer, W. F., & Jackson, D. N. (1990). Component analysis versus common factor-analysis - some further observations. *Multivariate Behavioral Research*, *25*, 97–114.

Widaman, K. F., & Reise, S. P. (1997). Exploring the measurement invariance of psychological instruments: Applications in the substance use domain. In K. J. Bryant, M. Windle, & S. G. West (Eds.), *The science of prevention: Methodological advances from alcohol and substance abuse research* (pp. 281–324). Washington, DC: American Psychological Association.

Yesavage, J. A., Bruik, T. L., & Rose, T. L. (1983). Development and validation of a geriatric depression screening scale: A preliminary report. *Journal of Psychiatric Research*, *17*, 37–49.
