# Supplementary material for: The Current and Retrospective Cognitive Reserve (2CR) survey and its relationship with cognitive and mood measures
Source: Eur J Ageing. 2023 Jun 14;20(1):23. doi: 10.1007/s10433-023-00766-x (PMC10267060; doi:10.1007/s10433-023-00766-x)
Supplement: Supplementary file 2 — Additional file 2. 2CR survey protocol. [file 10433_2023_766_MOESM2_ESM.docx]

**Appendix A.**

**Current and Retrospective Cognitive Reserve survey (2CR)**

*Date:*

*Code/ID:*

*Sex (M/F): Date of birth: Age:*

**The following questions refer to different aspects of your life. After each question you will find a series of alternative answers. Please fill in the option that best represents your choice, by marking the corresponding box with an "X".**

**EDUCATION**

1. What is the highest level of education you achieved?

| Pre-school | Elementary school | Middle school | High  school | Bachelor’s Degree | Master’s degree | Doctorate  (PhD, MD) |
| --- | --- | --- | --- | --- | --- | --- |
|  |  |  |  |  |  |  |

1. How many years of schooling (including pre-school) have you completed? _____

**OCCUPATION**

1. What type of work have you mostly done in life?

(Think about the occupation you had for the longest period of time).

| Manual, non-qualified occupation | Manually skilled, qualification required | Non-manual, technical qualification required | Professional or freelance (with university degree) | Director or manager |
| --- | --- | --- | --- | --- |
|  |  |  |  |  |

**FINANCIAL WELLBEING**

1. How difficult is it for you to get through the month financially?

| Not difficult at all | Not very difficult | Difficult | Very difficult | Extremely difficult |
| --- | --- | --- | --- | --- |
|  |  |  |  |  |

**FAMILY ENGAGEMENT**

1. Are you married/ Do you have a partner? YES – NO
2. Do you have any children? YES – NO
   1. If yes, how many children do you have?
3. Do you have any grandchildren? YES – NO
   1. If yes, how many grandchildren do you have?

**Partnership quality**

1. Partner’s status: The following statements concern your current partner (if you have a partner). For each statement, indicate your degree of agreement.
   1. My partner has many **interests** that keep him/her engaged (busy):

| Strongly disagree | Disagree | Neither agree nor disagree | Agree | Strongly agree |
| --- | --- | --- | --- | --- |
|  |  |  |  |  |

1. My partner, overall, is in **good health**:

| Strongly disagree | Disagree | Neither agree nor disagree | Agree | Strongly agree |
| --- | --- | --- | --- | --- |
|  |  |  |  |  |

1. My partner is often in **a good mood**:

| Strongly disagree | Disagree | Neither agree nor disagree | Agree | Strongly agree |
| --- | --- | --- | --- | --- |
|  |  |  |  |  |

1. My partner still has a **good “mind”** (he/she has no memory or attention difficulties):

| Strongly disagree | Disagree | Neither agree nor disagree | Agree | Strongly agree |
| --- | --- | --- | --- | --- |
|  |  |  |  |  |

**Connectivity logistics**

1. **At present**, how often do you do the following activities to spend time or keep in touch with family and close friends?

|  | Never | Seldom (Yearly) | Sometimes  (Monthly) | Often  (Weekly) | Always  (Daily) |
| --- | --- | --- | --- | --- | --- |
| 1. Drive a car or take public transportation |  |  |  |  |  |
| 1. Use a computer, telephone, smart phone, or tablet to communicate |  |  |  |  |  |

**LEISURE ACTIVITY**

**Recreational exercise**

1. **At present,** how often do you practice the following recreational exercise activities?

|  | Never | Seldom (Yearly) | Sometimes  (Monthly) | Often  (Weekly) | Always  (Daily) |
| --- | --- | --- | --- | --- | --- |
| 1. Exercise at the gym |  |  |  |  |  |
| 1. Dancing |  |  |  |  |  |
| 1. Swimming or water aerobics |  |  |  |  |  |

*Other aerobic exercise (e.g., walking, cycling):* ______________________________________

**Creative expression**

1. **At present,** how often do you practice the following creative activities?

|  | Never | Seldom (Yearly) | Sometimes  (Monthly) | Often  (Weekly) | Always  (Daily) |
| --- | --- | --- | --- | --- | --- |
| 1. Playing a musical instrument |  |  |  |  |  |
| 1. Writing |  |  |  |  |  |
| 1. Painting or drawing |  |  |  |  |  |

*Other creative expression activity:* ______________________________________

**Intellectual stimulation**

1. **At present**, how often do you practice the following leisure activities?

|  | Never | Seldom (Yearly) | Sometimes  (Monthly) | Often  (Weekly) | Always  (Daily) |
| --- | --- | --- | --- | --- | --- |
| 1. Reading |  |  |  |  |  |
| 1. Playing chess |  |  |  |  |  |
| 1. Crosswords or other puzzles |  |  |  |  |  |

*Other games requiring strategy/reasoning:* ______________________________________

**SOCIAL ENGAGEMENT**

**Volunteering**

1. **At present,** how often do you perform volunteer work in the following settings?

|  | Never | Seldom (Yearly) | Sometimes  (Monthly) | Often  (Weekly) | Always  (Daily) |
| --- | --- | --- | --- | --- | --- |
| 1. Hospitals or personal care facilities |  |  |  |  |  |
| 1. Schools or other facilities dedicated to young people or children |  |  |  |  |  |
| 1. Any other association with voluntary or charity aims * |  |  |  |  |  |

*___________________________________________________________

**Associations**

1. **At present,** how often do you participate socially in the following settings?

|  | Never | Seldom (Yearly) | Sometimes  (Monthly) | Often  (Weekly) | Always  (Daily) |
| --- | --- | --- | --- | --- | --- |
| 1. Senior clubs, reading groups, associations for the elderly |  |  |  |  |  |
| 1. Political associations or groups |  |  |  |  |  |
| 1. Clubs, groups or associations of professionals (specify*) |  |  |  |  |  |

*___________________________________________________________

**Public events**

1. **At present,** how often do you practice the following activities?

|  | Never | Seldom (Yearly) | Sometimes  (Monthly) | Often  (Weekly) | Always  (Daily) |
| --- | --- | --- | --- | --- | --- |
| 1. Go to the cinema and / or theater and / or concerts |  |  |  |  |  |
| 1. Go to exhibitions and / or museums |  |  |  |  |  |
| 1. Go to conferences, social events, public events |  |  |  |  |  |

**RELIGIOUS / SPIRITUAL ACTIVITY**

**Individual activities**

1. **At present**, how often do you perform the following religious / spiritual activities?

|  | Never | Seldom (Yearly) | Sometimes  (Monthly) | Often  (Weekly) | Always  (Daily) |
| --- | --- | --- | --- | --- | --- |
| 1. Prayer |  |  |  |  |  |
| 1. Meditation |  |  |  |  |  |

**Group activities**

1. **At present**, how often do you participate in the following religious / spiritual activities?

|  | Never | Seldom (Yearly) | Sometimes  (Monthly) | Often  (Weekly) | Always  (Daily) |
| --- | --- | --- | --- | --- | --- |
| 1. Rites/ceremonies |  |  |  |  |  |
| 1. Other church or temple community events (dinners, travel, etc.) |  |  |  |  |  |

**The following questions refer to different aspects of your lifestyle when you were younger (specify age range –i.e. *20-35/40* years of age–). After each question you will find a series of alternative answers. Fill in the option that best represent your choice by marking the corresponding box with an "X".**

**LEISURE ACTIVITY**

**Recreational exercise**

1. **When you were young,** how often did you practice the following recreational exercise activities?

|  | Never | Seldom (Yearly) | Sometimes  (Monthly) | Often  (Weekly) | Always  (Daily) |
| --- | --- | --- | --- | --- | --- |
| 1. Exercise at the gym |  |  |  |  |  |
| 1. Dancing |  |  |  |  |  |
| 1. Swimming or water aerobics |  |  |  |  |  |

*Other aerobic exercise (e.g., walking, cycling):* ______________________________________

**Creative expression**

1. **When you were young,** how often did you practice the following creative activities?

|  | Never | Seldom (Yearly) | Sometimes  (Monthly) | Often  (Weekly) | Always  (Daily) |
| --- | --- | --- | --- | --- | --- |
| 1. Playing a musical instrument |  |  |  |  |  |
| 1. Writing |  |  |  |  |  |
| 1. Painting or drawing |  |  |  |  |  |

*Other creative expression activity:* ______________________________________

**Public events**

1. **When you were young,** how often did you practice the following activities?

|  | Never | Seldom (Yearly) | Sometimes  (Monthly) | Often  (Weekly) | Always  (Daily) |
| --- | --- | --- | --- | --- | --- |
| 1. Go to the cinema and / or theater and / or concerts |  |  |  |  |  |
| 1. Go to exhibitions and / or museums |  |  |  |  |  |
| 1. Go to conferences, social events, public events |  |  |  |  |  |

**SOCIAL ENGAGEMENT**

**Volunteering**

1. **When you were young,** how often did you do volunteer work in the following settings?

|  | Never | Seldom (Yearly) | Sometimes  (Monthly) | Often  (Weekly) | Always  (Daily) |
| --- | --- | --- | --- | --- | --- |
| 1. Hospitals or personal care facilities |  |  |  |  |  |
| 1. Schools or other facilities dedicated to young people or children |  |  |  |  |  |
| 1. Any other association with voluntary or charity aims* |  |  |  |  |  |

*___________________________________________________________

**Associations**

1. **When you were young,** how often did you participate socially in the following settings?

|  | Never | Seldom (Yearly) | Sometimes  (Monthly) | Often  (Weekly) | Always  (Daily) |
| --- | --- | --- | --- | --- | --- |
| 1. Special interest or hobbyist clubs, reading groups |  |  |  |  |  |
| 1. Political associations or groups |  |  |  |  |  |
| 1. Clubs, groups or associations of professionals (specify*) |  |  |  |  |  |

*___________________________________________________________

**Connectivity logistics**

1. **When you were young**, how often did you do the following to carry out volunteer and association-related activities?

|  | Never | Seldom (Yearly) | Sometimes  (Monthly) | Often  (Weekly) | Always  (Daily) |
| --- | --- | --- | --- | --- | --- |
| 1. Drive a car or take public transportation |  |  |  |  |  |
| 1. Use a computer, telephone, smart phone, or tablet to communicate |  |  |  |  |  |

**RELIGIOUS / SPIRITUAL ACTIVITY**

**Individual activities**

1. **When you were young**, how often did you perform the following religious / spiritual activities?

|  | Never | Seldom (Yearly) | Sometimes  (Monthly) | Often  (Weekly) | Always  (Daily) |
| --- | --- | --- | --- | --- | --- |
| 1. Prayer |  |  |  |  |  |
| 1. Meditation |  |  |  |  |  |

**Group activities**

1. **When you were young**, how often did you participate in the following religious / spiritual activities?

|  | Never | Seldom (Yearly) | Sometimes  (Monthly) | Often  (Weekly) | Always  (Daily) |
| --- | --- | --- | --- | --- | --- |
| 1. Rites/ceremonies |  |  |  |  |  |
| 1. Other church or temple community events (dinners, travel, etc.) |  |  |  |  |  |

**Riserva Cognitiva Attuale e Passata (2RC)**

*Codice partecipante*: ____________ *data somministrazione*: __ / __ / ____

*Data di nascita:* __ / __ / ____ *Età: ____ Genere: M F*

**Gentile partecipante, qui di seguito troverà una serie di domande relative a diversi aspetti della sua vita. Dopo ogni domanda troverà una serie di alternative di risposta. Le chiediamo di selezionare l’alternativa che meglio rispecchia la sua scelta, mettendo una crocetta sulla casella corrispondente.**

# SCOLARITÀ

1. Qual è il più alto grado di istruzione che ha raggiunto?

| Scuola dell’infanzia | Elementare | Media | Superiore | Laurea triennale | Laurea magistrale | Dottorato di ricerca |
| --- | --- | --- | --- | --- | --- | --- |

1. Quanti anni di scuola (incluso l’asilo) hai completato? ___

# OCCUPAZIONE

1. Che tipo di lavoro ha svolto nella sua vita? (Pensi al lavoro che ha svolto per più tempo)

| Manuale non qualificato | Manuale  qualificato | Qualificato tecnico, non manuale (es. impiegato) | Professionale o libero professionista (con laurea) | Direttore o manager |
| --- | --- | --- | --- | --- |

# SITUAZIONE FINANZIARIA

1. Quanto è difficoltoso per lei arrivare a fine mese economicamente?

| Per niente | Poco | Abbastanza | Molto | Moltissimo |
| --- | --- | --- | --- | --- |

# FAMIGLIA E COINVOLGIMENTO FAMILIARE

1. È sposato/a o ha un/a compagno/a? SI - NO

**6**. Ha figli? SI - NO

a) Se si, quanti? ______

**7**. Ha nipoti? SI - NO

a) Se si, quanti? ______

**Qualità delle relazioni**

**8.** Di seguito trova una serie di affermazioni che riguardano il suo partner/compagno-a. Per ognuna di queste indichi il suo grado d’accordo.

1. Il mio partner/compagno-a ha vari ​**interessi** ​che lo impegnano:

| In completo disaccordo | In disaccordo | Né d’accordo né in disaccordo | D’accordo | Completamente d’accordo |
| --- | --- | --- | --- | --- |

1. Il mio partner/compagno-a gode, in generale, di ​**buona salute**​:

| In completo disaccordo | In disaccordo | Né d’accordo né in disaccordo | D’accordo | Completamente d’accordo |
| --- | --- | --- | --- | --- |

1. Il mio partner/compagno-a è spesso di ​**buon umore**​:

| In completo disaccordo | In disaccordo | Né d’accordo né in disaccordo | D’accordo | Completamente d’accordo |
| --- | --- | --- | --- | --- |

1. Il mio partner/compagno-a ha ancora una buona “mente” (non ha difficoltà di memoria, o di attenzione etc.):

| In completo disaccordo | In disaccordo | Né d’accordo né in disaccordo | D’accordo | Completamente d’accordo |
| --- | --- | --- | --- | --- |

**Mezzi per rimanere in contatto con la famiglia**

**9. Attualmente**, quanto spesso svolge le seguenti attività per poter passare del tempo e rimanere in contatto con familiari/amici cari?

|  | Mai | Raramente  (una volta all’anno) | Qualche volta  (una volta al mese) | Spesso  (una volta a settimana) | Sempre  (tutti i giorni) |
| --- | --- | --- | --- | --- | --- |
| 1. Guidare la macchina o prendere mezzi pubblici |  |  |  |  |  |
| 1. Usare il computer, il telefono, il cellulare o il tablet per comunicare |  |  |  |  |  |

# TEMPO LIBERO

# Attività fisica/ricreativa

**10. Attualmente,** quanto spesso pratica le seguenti attività fisiche (ricreative)?

|  | Mai | Raramente  (una volta all’anno) | Qualche volta  (una volta al mese) | Spesso  (una volta a settimana) | Sempre  (tutti i giorni) |
| --- | --- | --- | --- | --- | --- |
| 1. Ginnastica |  |  |  |  |  |
| 1. Ballo |  |  |  |  |  |
| 1. Nuoto (nuoto libero, acqua gym) |  |  |  |  |  |

*Altre attività fisiche/ricreative (es., camminare, andare in bicicletta):* ________________________________

# Espressione creativa

**11. Attualmente,** quanto spesso pratica le seguenti attività creative?

|  | Mai | Raramente  (una volta all’anno) | Qualche volta  (una volta al mese) | Spesso  (una volta a settimana) | Sempre  (tutti i giorni) |
| --- | --- | --- | --- | --- | --- |
| 1. Suonare uno strumento musicale |  |  |  |  |  |
| 1. Scrivere |  |  |  |  |  |
| 1. Dipingere o disegnare |  |  |  |  |  |

# *Altre attività creative:* ______________________________________

# Attività mentali

**12. Attualmente**, quanto spesso pratica le seguenti attività ricreative?

|  | Mai | Raramente  (una volta all’anno) | Qualche volta  (una volta al mese) | Spesso  (una volta a settimana) | Sempre  (tutti i giorni) |
| --- | --- | --- | --- | --- | --- |
| 1. Leggere |  |  |  |  |  |
| 1. Giocare a scacchi |  |  |  |  |  |
| 1. Cruciverba, giochi enigmistici |  |  |  |  |  |

*Altre attività/giochi che richiedono strategia/ragionamento:* _______________________________________

**COINVOLGIMENTO SOCIALE**

**Volontariato**

**13. Attualmente,** quanto spesso pratica attività di volontariato, nelle strutture di seguito riportate?

|  | Mai | Raramente  (una volta all’anno) | Qualche volta  (una volta al mese) | Spesso  (una volta a settimana) | Sempre  (tutti i giorni) |
| --- | --- | --- | --- | --- | --- |
| 1. Ospedali o strutture dedicate alla cura della persona |  |  |  |  |  |
| 1. Scuole o altre strutture dedicate a giovani o bambini |  |  |  |  |  |
| 1. Qualsiasi altra associazione ai fini di volontariato o carità* |  |  |  |  |  |

*____________________________________________________________________

# Partecipazione ad associazioni / club

**14. Attualmente,** quanto spesso pratica attività sociali, nelle strutture di seguito riportate?

|  | Mai | Raramente  (una volta all’anno) | Qualche volta  (una volta al mese) | Spesso  (una volta a settimana) | Sempre  (tutti i giorni) |
| --- | --- | --- | --- | --- | --- |
| 1. Club senior, gruppi di lettura, associazioni per anziani |  |  |  |  |  |
| 1. Associazioni o gruppi politici |  |  |  |  |  |
| 1. Circoli, gruppi o associazioni di professionisti (specificare il grado di coinvolgimento/ mansione svolta*) |  |  |  |  |  |

*_____________________________________________________________________________

# Eventi pubblici

**15. Attualmente,** quanto spesso pratica le seguenti attività?

|  | Mai | Raramente  (una volta all’anno) | Qualche volta  (una volta al mese) | Spesso  (una volta a settimana) | Sempre  (tutti i giorni) |
| --- | --- | --- | --- | --- | --- |
| 1. Andare al cinema e/o teatro e/o concerti |  |  |  |  |  |
| 1. Andare a mostre e/o musei |  |  |  |  |  |
| 1. Andare a conferenze, manifestazioni sociali, eventi pubblici |  |  |  |  |  |

# SPIRITUALITÀ

**Attività individuali**

**16. Attualmente,** quanto spesso pratica le seguenti attività religiose/spirituali?

|  | Mai | Raramente  (una volta all’anno) | Qualche volta  (una volta al mese) | Spesso  (una volta a settimana) | Sempre  (tutti i giorni) |
| --- | --- | --- | --- | --- | --- |
| 1. Pregare in privato |  |  |  |  |  |
| 1. Meditare |  |  |  |  |  |

**Attività collettive / di comunità**

**17. Attualmente,** quanto spesso partecipa alle seguenti attività religiose/spirituali?

|  | Mai | Raramente  (una volta all’anno) | Qualche volta  (una volta al mese) | Spesso  (una volta a settimana) | Sempre  (tutti i giorni) |
| --- | --- | --- | --- | --- | --- |
| 1. Riti / cerimonie |  |  |  |  |  |
| 1. Altri eventi della propria comunità religiosa (cene, viaggi etc) |  |  |  |  |  |

**Di seguito troverà una serie di domande relative al suo stile di vita quando era più giovane (specificare l’età, ad es. 20-35/40 anni). Dopo ogni domanda troverà una serie di alternative di risposta. Le chiediamo di selezionare l’alternativa che meglio rispecchia la sua scelta, mettendo una crocetta sulla casella corrispondente.**

# TEMPO LIBERO

# Attività fisica/ricreativa

**18. Da giovane,** quanto spesso praticava le seguenti attività fisiche (ricreative)?

|  | Mai | Raramente  (una volta all’anno) | Qualche volta  (una volta al mese) | Spesso  (una volta a settimana) | Sempre  (tutti i giorni) |
| --- | --- | --- | --- | --- | --- |
| 1. Ginnastica |  |  |  |  |  |
| 1. Ballo |  |  |  |  |  |
| 1. Nuoto (nuoto libero, acqua gym) |  |  |  |  |  |

*Altre attività fisiche/ricreative (es., camminare, andare in bicicletta):* ________________________

# Espressione creativa

**19. Da giovane,** quanto spesso praticava le seguenti attività creative?

|  | Mai | Raramente  (una volta all’anno) | Qualche volta  (una volta al mese) | Spesso  (una volta a settimana) | Sempre  (tutti i giorni) |
| --- | --- | --- | --- | --- | --- |
| 1. Suonare uno strumento musicale |  |  |  |  |  |
| 1. Scrivere |  |  |  |  |  |
| 1. Dipingere |  |  |  |  |  |

*Altre attività creative*: _____________________________________________________________

# Eventi pubblici

**20**. **Da giovane,** quanto spesso praticava le seguenti attività?

|  | Mai | Raramente  (una volta all’anno) | Qualche volta  (una volta al mese) | Spesso  (una volta a settimana) | Sempre  (tutti i giorni) |
| --- | --- | --- | --- | --- | --- |
| 1. Andare al cinema e/o teatro e/o concerti |  |  |  |  |  |
| 1. Andare a mostre e/o musei |  |  |  |  |  |
| 1. Andare a conferenze, manifestazioni sociali, eventi pubblici |  |  |  |  |  |

**COINVOLGIMENTO SOCIALE**

# Volontariato

**21. Da giovane,** quanto spesso praticava attività di volontariato, nelle strutture di seguito riportate?

|  | Mai | Raramente  (una volta all’anno) | Qualche volta  (una volta al mese) | Spesso  (una volta a settimana) | Sempre  (tutti i giorni) |
| --- | --- | --- | --- | --- | --- |
| 1. Ospedali o strutture dedicate alla cura della persona |  |  |  |  |  |
| 1. Scuole o altre strutture dedicate a giovani o bambini |  |  |  |  |  |
| 1. Qualsiasi altra associazione ai fini di volontariato o carità * |  |  |  |  |  |

*____________________________________________________________________

# Partecipazione ad associazioni / club

**22. Da giovane,** quanto spesso praticava attività sociali, nelle strutture di seguito riportate?

|  | Mai | Raramente  (una volta all’anno) | Qualche volta  (una volta al mese) | Spesso  (una volta a settimana) | Sempre  (tutti i giorni) |
| --- | --- | --- | --- | --- | --- |
| 1. Club senior, gruppi di lettura, associazioni per anziani |  |  |  |  |  |
| 1. Associazioni o gruppi politici |  |  |  |  |  |
| 1. Circoli, gruppi o associazioni di professionisti (specificare il grado di coinvolgimento/ mansione svolta*) |  |  |  |  |  |

*____________________________________________________________________

# Mezzi per svolgere attività

**23**. **Da giovane,** quanto spesso svolgeva le seguenti attività per praticare attività di volontariato e associazionismo?

|  | Mai | Raramente  (una volta all’anno) | Qualche volta  (una volta al mese) | Spesso  (una volta a settimana) | Sempre  (tutti i giorni) |
| --- | --- | --- | --- | --- | --- |
| 1. Guidare la macchina o prendere i mezzi pubblici |  |  |  |  |  |
| 1. Usare il computer, il telefono, il cellulare o il tablet per comunicare |  |  |  |  |  |

# SPIRITUALITÀ

**Attività individuali**

**24. Da giovane,** quanto spesso praticava le seguenti attività religiose/spirituali?

|  | Mai | Raramente  (una volta all’anno) | Qualche volta  (una volta al mese) | Spesso  (una volta a settimana) | Sempre  (tutti i giorni) |
| --- | --- | --- | --- | --- | --- |
| 1. Pregare in privato |  |  |  |  |  |
| 1. Meditare |  |  |  |  |  |

**Attività collettive / di comunità**

**25. Da giovane,** quanto spesso partecipava alle seguenti attività religiose/spirituali?

|  | Mai | Raramente  (una volta all’anno) | Qualche volta  (una volta al mese) | Spesso  (una volta a settimana) | Sempre  (tutti i giorni) |
| --- | --- | --- | --- | --- | --- |
| 1. Riti / cerimonie religiose |  |  |  |  |  |
| 1. Altri eventi della propria comunità religiosa (cene, viaggi etc) |  |  |  |  |  |

**Scoring Instructions**

Scoring is performed sequentially across the three levels of the CR framework. First, level-1 variables’ scores are calculated as a single number scaled 0-4. This is the most complicated step and is described further below. Next, level-2 scores (domain-specific CR scores) are calculated as averages of the corresponding level-1 scores. Finally, level-3 scores (global CR scores) are calculated as averages of the corresponding level-2 scores. The following instructions pertain to the specific calculations required at each level.

1. **Level-1 variables**’ scores correspond to one or more survey questions, as follows:

| **Level-1 Variable** | **Description** | **2CR Item(s)** |
| --- | --- | --- |
| ed_r | highest education level | 1 (*multiplied by 2/3*) |
| ys_r | years of schooling | 2 (*stratified for manual scoring*) |
| oc_c | occupational class | 3 |
| fw_c | financial wellbeing, current | 4 (*reverse scored*) |
| pq_c | partnership quality, current | 8a,8b,8c,8d |
| cl_c | connectivity logistics, current | 9a,9b |
| re_c | recreational exercise, current | 10a,10b,10c |
| ce_c | creative expression, current | 11a,11b,11c |
| is_c | intellectual stimulation, current | 12a,12b,12c |
| vl_c | volunteering, current | 13a,13b,13c |
| as_c | associations, current | 14a,14b,14c |
| pe_c | public events, current | 15a,15b,15c |
| ri_c | religious/spiritual activity (individual), current | 16a,16b |
| rg_c | religious/spiritual activity (group), current | 17a,17b |
| re_r | recreational exercise, retrospective | 18a,18b,18c |
| ce_r | creative expression, retrospective | 19a,19b,19c |
| pe_r | public events, retrospective | 20a,20b,20c |
| vl_r | volunteering, retrospective | 21a,21b,21c |
| as_r | associations, retrospective | 22a,22b,22c |
| cl_r | connectivity logistics, retrospective | 23a,23b |
| ri_r | religious/spiritual activity (individual), retrospective | 24a,24b |
| rg_r | religious/spiritual activity (group), retrospective | 25a,25b |

Responses to level-1 items are scaled 0–4, from left-to-right across response categories: e.g.,

| 0 | 1 | 2 | 3 | 4 |
| --- | --- | --- | --- | --- |
| Strongly disagree | Disagree | Neither agree nor disagree | Agree | Strongly agree |
| Never | Yearly | Monthly | Weekly | Daily |

Education level (item 1) spans seven (rather than five) response categories. Thus, education level is first scored 0-6, and these scores are then post-multiplied by 2/3 to arrive at a consistent scaling.

Years of schooling is assessed as a categorical variable. For manual scoring, this should be stratified into five ordinal responses (0-5) for consistent weighting with other level 1 variables. E.g., 0-5 years, 6-10 years, 11-15 years, 16-20 years, > 20 years.

Financial wellbeing (item 4) should be reverse scored (e.g., 0=4, 1=3, 2=3, 3=1, 4=0) prior to its inclusion in subsequent calculations.

For unmarried/unpartnered persons, scores for partnership quality items (8a, 8b, 8c, 8d) should be assigned values of 2 (“neither agree nor disagree”).

Scores for level-1 variables based on multiple items (parcels) are calculated as the average of the corresponding items. For example, the level-1 score for current intellectual simulation (is_c) is calculated as the mean of items 12a, 12b, and 12c.

- - - 1. **Level-2 variables**’ scores are calculated as averages of the corresponding level-1 variables’ scores:

| **Level-2 Variable** | **Description** | **Level-1 Variables** |
| --- | --- | --- |
| ses_c | socio-economic status, current | oc_c, fw_c |
| fam_c | family engagement, current | pq_c, cl_c |
| lei_c | leisure activity, current | re_c, ce_c, is_c |
| soc_c | social engagement, current | vl_c, as_c, pe_c |
| rel_c | religious/spiritual activity, current | ri_c, rg_c |
| ses_r | socio-economic status, retrospective | ed_r, ys_r |
| lei_r | leisure activity, retrospective | re_r, ce_r, pe_r |
| soc_r | social engagement, retrospective | vl_r, as_r, cl_r |
| rel_r | religious/spiritual activity, retrospective | ri_r, rg_r |

- - - 1. **Level-3** variables’ scores are calculated as averages of the corresponding level-2 variables’ scores:

| **Level-3 Variable** | **Description** | **Level-2 Variables** |
| --- | --- | --- |
| CR_c | cognitive reserve, current | ses_c, fam_c, lei_c, soc_c, rel_c |
| CR_r | cognitive reserve, retrospective | ses_r, lei_r, soc_r, rel_r |

Important note: Researchers may optionally exclude religious/spiritual activity items in calculations of global cognitive reserve scores (CR_c, CR_r). Religious practice was found in the initial test samples (during survey development) to be inversely related to cognitive performance but was also related to having fewer depressive symptoms. Religious practice items may function differentially across study populations. Hence, the decision of whether to include religious/spiritual activity in total CR scores should be made contingent on the specific research goals, and noted accordingly.
