# Supplementary material for: The Current and Retrospective Cognitive Reserve (2CR) survey and its relationship with cognitive and mood measures
Source: Eur J Ageing. 2023 Jun 14;20(1):23. doi: 10.1007/s10433-023-00766-x (PMC10267060; doi:10.1007/s10433-023-00766-x)
Supplement: Supplementary file 3 — Additional file 3. Mplus output. [file 10433_2023_766_MOESM3_ESM.docx]

**Supplemental Materials 2**

**The Current and Retrospective Cognitive Reserve (2CR) Survey and its relationship with cognitive and mood measures.**

**Mplus Output for Three-level 2CR model with CRc and CRr global factors.**

**MODEL FIT INFORMATION**

Number of Free Parameters 88

Loglikelihood

H0 Value -6215.143

H1 Value -6037.752

Information Criteria

Akaike (AIC) 12606.287

Bayesian (BIC) 12910.730

Sample-Size Adjusted BIC 12631.807

(n* = (n + 2) / 24)

Chi-Square Test of Model Fit

Value 354.783

Degrees of Freedom 187

P-Value 0.0000

RMSEA (Root Mean Square Error Of Approximation)

Estimate 0.062

90 Percent C.I. 0.052 0.072

Probability RMSEA <= .05 0.026

CFI/TLI

CFI 0.913

TLI 0.892

Chi-Square Test of Model Fit for the Baseline Model

Value 2150.677

Degrees of Freedom 231

P-Value 0.0000

SRMR (Standardized Root Mean Square Residual)

Value 0.067

**MODEL RESULTS**

Two-Tailed

Estimate S.E. Est./S.E. P-Value

CLEISURE BY

C_RECEXR 1.000 0.000 999.000 999.000

C_CRTEXP 0.697 0.118 5.900 0.000

C_MNTSTM 1.060 0.157 6.746 0.000

CSOCIAL BY

C_VOLUNT 1.000 0.000 999.000 999.000

C_ASSOCI 0.770 0.160 4.818 0.000

C_PUBEVN 2.005 0.340 5.898 0.000

CRELIGIO BY

C_RELIND 1.000 0.000 999.000 999.000

C_RELGRP 0.700 0.261 2.687 0.007

CSES BY

C_OCCCLA 1.000 0.000 999.000 999.000

C_FINANC 0.322 0.092 3.501 0.000

CFAM BY

C_QALPRT 1.000 0.000 999.000 999.000

C_CONNEC 1.839 0.468 3.926 0.000

RLEISURE BY

R_RECEXR 1.000 0.000 999.000 999.000

R_CRTEXP 0.815 0.122 6.665 0.000

R_PUBEVN 0.912 0.141 6.449 0.000

RSOCIAL BY

R_VOLUNT 1.000 0.000 999.000 999.000

R_ASSOCI 0.822 0.122 6.747 0.000

R_CONNEC 1.251 0.188 6.651 0.000

RRELIGIO BY

R_RELIND 1.000 0.000 999.000 999.000

R_RELGRP 0.857 0.272 3.152 0.002

RSES BY

R_EDULVL 1.000 0.000 999.000 999.000

R_SCHYRS 3.730 0.150 24.890 0.000

CR_CRNT BY

CLEISURE 1.000 0.000 999.000 999.000

CSOCIAL 0.755 0.153 4.927 0.000

CRELIGIO 0.377 0.171 2.199 0.028

CSES 0.938 0.209 4.484 0.000

CFAM 0.644 0.156 4.133 0.000

CR_RTRO BY

RLEISURE 1.000 0.000 999.000 999.000

RSOCIAL 0.784 0.151 5.194 0.000

RRELIGIO 0.574 0.170 3.367 0.001

RSES 1.300 0.215 6.052 0.000

CLEISURE WITH

RLEISURE 0.009 0.023 0.391 0.696

CSOCIAL WITH

RSOCIAL -0.009 0.013 -0.724 0.469

CRELIGIO WITH

RRELIGIO 0.491 0.185 2.655 0.008

CSES WITH

RSES 0.430 0.079 5.422 0.000

CR_RTRO WITH

CR_CRNT 0.192 0.042 4.533 0.000

C_CRTEXP WITH

R_CRTEXP 0.212 0.033 6.458 0.000

C_RECEXR WITH

R_RECEXR 0.186 0.043 4.329 0.000

C_PUBEVN WITH

R_PUBEVN 0.072 0.031 2.320 0.020

C_VOLUNT WITH

R_VOLUNT 0.150 0.035 4.247 0.000

C_ASSOCI WITH

R_ASSOCI 0.072 0.022 3.231 0.001

C_RELIND WITH

R_RELIND 0.244 0.176 1.384 0.166

C_RELGRP WITH

R_RELGRP 0.275 0.115 2.397 0.017

C_CONNEC WITH

R_CONNEC 0.181 0.067 2.697 0.007

Intercepts

C_OCCCLA 1.540 0.076 20.359 0.000

C_FINANC 3.055 0.057 53.214 0.000

C_RELIND 1.904 0.071 26.858 0.000

C_RELGRP 1.417 0.073 19.435 0.000

C_MNTSTM 1.451 0.052 28.031 0.000

C_CRTEXP 0.665 0.042 15.804 0.000

C_RECEXR 0.796 0.053 15.146 0.000

C_VOLUNT 0.552 0.051 10.714 0.000

C_ASSOCI 0.387 0.039 9.941 0.000

C_PUBEVN 1.243 0.054 22.998 0.000

C_QALPRT 2.822 0.058 48.401 0.000

C_CONNEC 2.735 0.078 35.213 0.000

R_SCHYRS 10.183 0.263 38.740 0.000

R_EDULVL 3.404 0.074 46.000 0.000

R_RELIND 1.849 0.072 25.545 0.000

R_RELGRP 2.238 0.079 28.326 0.000

R_RECEXR 1.574 0.063 24.813 0.000

R_CRTEXP 0.984 0.053 18.514 0.000

R_PUBEVN 1.615 0.053 30.615 0.000

R_VOLUNT 0.556 0.053 10.420 0.000

R_ASSOCI 0.397 0.046 8.626 0.000

R_CONNEC 0.843 0.069 12.276 0.000

Variances

CR_CRNT 0.201 0.050 4.020 0.000

CR_RTRO 0.252 0.064 3.943 0.000

Residual Variances

C_OCCCLA 0.550 0.215 2.556 0.011

C_FINANC 0.692 0.067 10.261 0.000

C_RELIND 0.316 0.316 1.001 0.317

C_RELGRP 0.825 0.175 4.703 0.000

C_MNTSTM 0.403 0.046 8.817 0.000

C_CRTEXP 0.318 0.033 9.703 0.000

C_RECEXR 0.447 0.047 9.606 0.000

C_VOLUNT 0.508 0.050 10.207 0.000

C_ASSOCI 0.288 0.028 10.263 0.000

C_PUBEVN 0.221 0.047 4.684 0.000

C_QALPRT 0.594 0.069 8.647 0.000

C_CONNEC 1.072 0.154 6.944 0.000

R_SCHYRS 0.456 0.494 0.923 0.356

R_EDULVL 0.153 0.038 4.012 0.000

R_RELIND 0.363 0.270 1.348 0.178

R_RELGRP 0.830 0.208 3.998 0.000

R_RECEXR 0.605 0.072 8.373 0.000

R_CRTEXP 0.438 0.053 8.218 0.000

R_PUBEVN 0.369 0.050 7.356 0.000

R_VOLUNT 0.377 0.051 7.405 0.000

R_ASSOCI 0.302 0.037 8.056 0.000

R_CONNEC 0.651 0.083 7.826 0.000

CLEISURE 0.001 0.024 0.031 0.975

CSOCIAL 0.001 0.012 0.103 0.918

CRELIGIO 0.837 0.322 2.597 0.009

CSES 0.618 0.238 2.599 0.009

CFAM 0.016 0.034 0.465 0.642

RLEISURE 0.089 0.045 1.952 0.051

RSOCIAL 0.136 0.038 3.588 0.000

RRELIGIO 0.785 0.276 2.838 0.005

RSES 0.708 0.092 7.670 0.000
